# Leveraging genomic diversity for discovery in an electronic health record linked biobank: the UCLA ATLAS Community Health Initiative

**DOI:** 10.1186/s13073-022-01106-x

**Published:** 2022-09-09

**Authors:** Ruth Johnson, Yi Ding, Vidhya Venkateswaran, Arjun Bhattacharya, Kristin Boulier, Alec Chiu, Sergey Knyazev, Tommer Schwarz, Malika Freund, Lingyu Zhan, Kathryn S. Burch, Christa Caggiano, Brian Hill, Nadav Rakocz, Brunilda Balliu, Christopher T. Denny, Jae Hoon Sul, Noah Zaitlen, Valerie A. Arboleda, Eran Halperin, Sriram Sankararaman, Manish J. Butte, UCLA Precision Health Data Discovery Repository Working Group, UCLA Precision Health ATLAS Working Group, Clara Lajonchere, Daniel H. Geschwind, Bogdan Pasaniuc

**Affiliations:** 1 Department of Computer Science, University of California, Los Angeles, Los Angeles, CA 90095 USA; 2 Department of Pathology and Laboratory Medicine, David Geffen School of Medicine, University of California, Los Angeles, Los Angeles, CA 90095 USA; 3 Bioinformatics Interdepartmental Program, University of California, Los Angeles, Los Angeles, CA 90095 USA; 4 Department of Oral Biology, School of Dentistry, University of California, Los Angeles, Los Angeles, CA 90095 USA; 5 Institute for Quantitative and Computational Biosciences, David Geffen School of Medicine, University of California, Los Angeles, Los Angeles, CA 90095 USA; 6 Department of Medicine, Division of Cardiology, University of California, Los Angeles, Los Angeles, CA 90095 USA; 7 Department of Human Genetics, David Geffen School of Medicine, University of California, Los Angeles, Los Angeles, CA 90095 USA; 8 Department of Genetics, Stanford School of Medicine, Stanford, CA 94305 USA; 9 Molecular Biology Institute, David Geffen School of Medicine, University of California, Los Angeles, Los Angeles, CA 90095 USA; 10 Program in Neurogenetics, Department of Neurology, David Geffen School of Medicine, University of California, Los Angeles, Los Angeles, CA 90095 USA; 11 Department of Computational Medicine, David Geffen School of Medicine, University of California, Los Angeles, Los Angeles, CA 90095 USA; 12 Division of Hematology/Oncology, Department of Pediatrics, Gwynne Hazen Cherry Memorial Laboratories, University of California, Los Angeles, Los Angeles, CA 90095 USA; 13 Molecular Biology Institute, University of California, Los Angeles, Los Angeles, CA 90095 USA; 14 Jonsson Comprehensive Cancer Center, University of California, Los Angeles, Los Angeles, CA 90095 USA; 15 Department of Psychiatry and Biobehavioral Sciences, University of California, Los Angeles, Los Angeles, CA 90095 USA; 16 Department of Anesthesiology and Perioperative Medicine, David Geffen School of Medicine, University of California, Los Angeles, Los Angeles, CA 90095 USA; 17 Department of Pediatrics, David Geffen School of Medicine, University of California, Los Angeles, Los Angeles, CA 90095 USA; 18 Institute of Precision Health, University of California, Los Angeles, Los Angeles, CA 90095 USA

**Keywords:** Electronic health records, Biobank, Genetic ancestry, Genome-wide association studies, Phenome-wide association studies

## Abstract

**Background:**

Large medical centers in urban areas, like Los Angeles, care for a diverse patient population and offer the potential to study the interplay between genetic ancestry and social determinants of health. Here, we explore the implications of genetic ancestry within the University of California, Los Angeles (UCLA) ATLAS Community Health Initiative—an ancestrally diverse biobank of genomic data linked with de-identified electronic health records (EHRs) of UCLA Health patients (*N*=36,736).

**Methods:**

We quantify the extensive continental and subcontinental genetic diversity within the ATLAS data through principal component analysis, identity-by-descent, and genetic admixture. We assess the relationship between genetically inferred ancestry (GIA) and >1500 EHR-derived phenotypes (phecodes). Finally, we demonstrate the utility of genetic data linked with EHR to perform ancestry-specific and multi-ancestry genome and phenome-wide scans across a broad set of disease phenotypes.

**Results:**

We identify 5 continental-scale GIA clusters including European American (EA), African American (AA), Hispanic Latino American (HL), South Asian American (SAA) and East Asian American (EAA) individuals and 7 subcontinental GIA clusters within the EAA GIA corresponding to Chinese American, Vietnamese American, and Japanese American individuals. Although we broadly find that self-identified race/ethnicity (SIRE) is highly correlated with GIA, we still observe marked differences between the two, emphasizing that the populations defined by these two criteria are not analogous. We find a total of 259 significant associations between continental GIA and phecodes even after accounting for individuals’ SIRE, demonstrating that for some phenotypes, GIA provides information not already captured by SIRE. GWAS identifies significant associations for liver disease in the 22q13.31 locus across the HL and EAA GIA groups (HL *p*-value=2.32×10^−16^, EAA *p*-value=6.73×10^−11^). A subsequent PheWAS at the top SNP reveals significant associations with neurologic and neoplastic phenotypes specifically within the HL GIA group.

**Conclusions:**

Overall, our results explore the interplay between SIRE and GIA within a disease context and underscore the utility of studying the genomes of diverse individuals through biobank-scale genotyping linked with EHR-based phenotyping.

**Supplementary Information:**

The online version contains supplementary material available at 10.1186/s13073-022-01106-x.

## Background

Linking electronic health records (EHRs) to patient genomic data within biobanks in a de-identified fashion has the potential to significantly advance genomic discoveries and precision medicine efforts (e.g., population screening, identifying drug targets) [1–4]. However, the underrepresentation of minoritized populations in biomedical research [5–11] raises concerns that advancements in precision medicine may widen disparities in access to high-quality health care [12–14]. For example, European-ancestry individuals constitute approximately 16% of the global population, yet account for almost 80% of all genome-wide association study (GWAS) participants [13]. As a direct result of this imbalance, existing methods to predict disease risk from genetics (e.g., polygenic risk scores) are vastly inaccurate in individuals of non-European ancestry [13, 15] thus forming a barrier for advancing genomic medicine to benefit patients of all ancestries.

The University of California, Los Angeles (UCLA) Health medical system is located in Los Angeles, one of the most ethnically diverse cities in the world. There is no ethnic majority: 48.5% of Los Angeles residents self-identify as Hispanic or Latino, 11.6% as Asian, and 8.9% as Black or African American; additionally, 37% of Los Angeles residents are neither U.S. nationals, nor U.S. citizens at birth [16]. Therefore, the UCLA Health patient population and the availability of digital health data captured in EHRs from a single medical system presents a unique opportunity to increase the inclusion of underrepresented minorities in biomedical research. In this study, we investigate the role of genetic ancestry in a disease context within the UCLA ATLAS Community Health Initiative (or ATLAS for brevity), a biobank embedded within the UCLA Health medical system composed of de-identified, EHR-linked genomic data from a diverse patient population [17, 18]. The current initiative aims to collect genomic data from over 150,000 individuals; currently this consists of *N*=36,736 individuals genotyped at *M*=667,191 SNPs genome-wide using the Illumina global screening array (GSA) [19] and then imputed to >8 million SNPs using a multi-ancestry imputation panel (TOPMed Freeze5 [20]). A detailed description describing the recruitment, consent process, sample collection, and genotype and phenotype quality control are discussed in prior works [17, 18, 21].

The EHR contains a de-identified extract of medical records (billing codes, laboratory values, etc.) as well as demographic information such as self-identified race and ethnicity information. It is important to note that self-identified race and ethnicity (SIRE) represent social constructs that capture shared values, cultural norms, and behaviors of subgroups [22] that are distinct concepts from genetic ancestry which refers to the ancestral history of one’s genome. This difference is even more relevant for individuals self-describing as multi-racial (and/or admixed) where genetic ancestry bears little correlation to SIRE [23, 24]. Understanding the interplay of genetic factors (such as genetic ancestry) with social determinants of health (as inferred from self-reports) is still mired in the confounding overlaps between race, socioeconomic status, and disease, but serves as a critical step in mapping and predicting disease risk across individuals of all ancestries.

In this work, we leverage the unique genomic diversity of our single-center cohort to explore the interconnected effects of self-identified race/ethnicity and genetic ancestry on clinical phenotypes. We cluster individuals by genetically inferred ancestry (GIA) within the EHR-linked biobank, systematically construct phenotypes from EHR, and compute disease associations using multi-ancestry pipelines for both genome-wide and phenome-wide association studies (PheWAS). We find that genetically derived and self-identified information yield distinct subpopulations, emphasizing the distinction between GIA and SIRE. We leverage genetic and self-identified data to find extensive variation of subcontinental ancestry within ATLAS across European American (EA), East Asian American (EAA), Hispanic Latino American (HL), and African American (AA) GIA groups. For example, we find clusters of individuals with recent inferred ancestry from Filipino, Chinese, Japanese, and Korean ancestries among the EAA cluster. Such subcontinental clusters also stratify individuals according to disease groups thus emphasizing their utility in biomedical research. We perform both ancestry-specific GWAS and meta-analyses across GIA groups and recapitulate known genomic risk regions. We perform PheWAS on significant regions and describe genetic associations for liver-related phenotypes in multiple ancestry groups as well as associations with neurologic and neoplastic phenotypes that are associated exclusively in the HL GIA group. These results underscore how the utility of large-scale genetic analyses and deep phenotyping in diverse populations can make substantial medical contributions for population health.

## Methods

### Study population

The UCLA Health System includes two hospitals (520 and 281 inpatient beds) and 210 primary and specialty outpatient locations predominantly located in Los Angeles County. The UCLA Data Discovery Repository (DDR) contains de-identified patient EHRs that have been collected since March 2, 2013, under the auspices of the UCLA Health Office of Health Informatics Analytics and the UCLA Institute of Precision Health. Currently, the DDR contains longitudinal records for more than 1.5 million patients (inpatient and outpatient), including basic patient information (height, weight, gender), diagnosis codes, laboratory tests, medications, prescriptions, hospital admissions, and procedures. The UCLA ATLAS Community Health Initiative includes the EHR-linked biobank within the UCLA Health System. Currently, there are more than 37,000 genotyped participants with their de-identified EHR linked through the DDR. Participation is voluntary and privacy is protected by de-identifying the samples. Additional information regarding recruitment, consent, sample processing, and quality control pipelines can be found in previous work [17, 18, 21]. Patient Recruitment and Sample Collection for Precision Health Activities at UCLA is an approved study by the UCLA Institutional Review Board (UCLA IRB). IRB#17-001013.

### Self-identified demographic information

Self-identified demographic information is collected as a part of clinical care which is then translated to the EHR. Participants self-identify race and ethnicity via two distinct drop-down fields where there are pre-determined multiple-choice fields for race and ethnicity (see Additional file 2: Table S1, S2 for full list containing exact terminology). At this time, only one selection from each category can be chosen as a patient’s primary race and ethnicity [25]. We group together race/ethnicity pairings to form “self-identified race/ethnicity” (SIRE) groupings (Additional file 2: Table S3). Patients also report their “Preferred Language” from pre-determined multiple-choice fields within the EHR. See the section “Notes on terminology and naming conventions” for a more detailed discussion of terminology used for SIREs.

### Notes on terminology and naming conventions

In this section, we explicitly discuss the origin of the terminology and naming conventions used throughout this manuscript with respect to genetic ancestry, race, and ethnicity. We refer to Peterson et al. [26] for a more comprehensive description of terms for GWAS in ancestrally diverse populations.

The term “genetic ancestry” refers to the characterization of the population(s) from which an individual is descended and describes the genetic relationship implied by shared, large segments of genomic DNA between an individual and these ancestors [27]. Throughout this work, we reserve this term to describe individuals with information about the origin of their recent biological ancestors. For instance, we treat populations represented in genetic reference panels (e.g., 1000 Genomes Project [28, 29]) as instances of genetic ancestry since the information describing the origin of the recent biological ancestors represented in the samples is known.

Much of this work involves inferring the genetic ancestry information for a set of individuals. We introduce the term “genetically inferred ancestry (GIA)” to describe the genetic characterization of individuals within a group who likely share recent biological ancestors as inferred by a method of choice. We emphasize that GIA differs from genetic ancestry in that GIA depends on the inference method (e.g., clustering) and choice of reference data (e.g., 1000 Genomes).

The terms “Native American genetic ancestry” and “Native American GIA” refer to ancestry and/or recent biological ancestors from individuals originating from indigenous groups originally from North America, Central American, and South America. The term “Native American race” refers to the definition used by the US Census, “ a person having origins in any of the original peoples of North and South America (including Central America) and who maintains tribal affiliation or community attachment” [30]. We recognize that individuals in this group may prefer other terms such as “American Indians.” To be clear, identification of subjects as Native American GIA is not meant to imply a tribal status.

In the context of this work, the term “African genetic ancestry” describes individuals whose recent biological ancestors originated from the continent of Africa. “African American (AA) GIA” refers to an admixed group of individuals within the USA who have recent biological ancestors inferred to be of African ancestry and thus have partial or total ancestry originating from Africa. The term “Admixed American ancestry” refers to those with recent biological ancestors from European, African, and Native American ancestries that achieved admixture in North America, Central America, and South America. Thus, Admixed American ancestry contains global proportions of all three ancestry groups. “Hispanic Latino American (HL) GIA” refers to the group of admixed individuals within the USA whose recent biological ancestors were inferred to be individuals of Admixed American ancestry. “European ancestry” refers to individuals with recent biological ancestors with origins in continental Europe. “European American (EA) GIA” refers to individuals within the USA with recent biological ancestors inferred to be of European ancestry, thus, partial or total ancestry originating from Europe. “East Asian ancestry” and “South Asian ancestry” refers to individuals with recent biological ancestors from East Asia and South Asia respectively. “East Asian American (EAA) GIA” and “South Asian American (SAA) GIA” refers to individuals within the USA with recent biological ancestors inferred to be of East Asian ancestry or South Asian ancestry.

We disapprove that the term “White/Caucasian” is a preset multiple-choice option under the race field within the medical records and renounce its usage due to its erroneous origins and historically racist implications. We strongly discourage the connection of the term “Caucasian” with the discussion of race, a social construct separate from biology, and emphasize that the term does not have biological implications [31]. For subsequent analyses presented in this work, we use “White” to refer to the “White/Caucasian” category. Although this terminology is still built into the language of many documents and surveys, such as EHRs, we make this change to avoid perpetuating its usage within the field.

### Basic genotype quality control

Bio-samples collected from the UCLA ATLAS Community Health Initiative in the form of blood samples were de-identified and then processed for DNA extraction and genotyping. We utilized a “frozen snapshot” of ATLAS data composed of all samples processed up to 6/18/2021. ATLAS participants (*N*=36,779) were genotyped using a custom genotyping array constructed from the Global Screening Array with the multi-disease drop-in panel [19] under the GRCh38 assembly. Overall, the array measured 700,079 sites for capturing single-nucleotide polymorphisms (SNPs) and short insertions and deletions (indels).

We filtered out poor-quality markers by removing unmapped SNPs, SNPs with >5% missingness, and strand-ambiguous SNPs (*M* = 19,313 variants removed). We excluded samples with missingness >5% (*N*=1 individual removed). We identified duplicate individuals (or identical twins/triplets, etc.) using KING 2.2.2 [32] (“--duplicate”) and removed the individual with the lowest missing rate from each pair (*N*=42 individuals removed). All quality control steps were conducted using PLINK 1.9 [33]. Following sample- and variant-level quality control, *M*=667,191 genotyped SNPs remained across *N*=36,736 individuals for downstream analyses. All subsequent genetic analyses in this paper utilize this QC’d set of genotypes. Additional steps of QC may be conducted before running specific analyses, as described in more detail below. A summary of the sample sizes and sets of SNPs used in subsequent analyses is described in Additional file 8: Table S14. We refer to our previous work for a more thorough description of the quality control pipelines constructed for ATLAS [17].

### Genotype imputation

After performing array-level genotype quality control, the PLINK-formatted genotypes were converted to VCF format and uploaded to the Michigan Imputation Server [34]. On a variant level, the server removed the variant if it was not an A, C, G, T allele, monomorphic, a duplicate, an allele mismatch between the reference panel and provided data, an insertion-deletion, or if the SNP call rate is less than 90%. The server will additionally correct for any necessary strand flips or allele switches needed to match the reference panel. The server additionally phases the data using Eagle v2.4 [35], and imputation is performed against the TOPMed Freeze5 imputation panel [20] using minimac4 [36]. In summary, the explicit parameters used on the server are “TOPMed Freeze5” for the reference panel, “GRCh38/hg38” for the array build, “off” for the rsq filter, “Eagle v2.4” for phasing, no QC frequency check, and “quality control & imputation” for the mode. After we filtered by *R*^2^>0.90 and MAF>1%, the final set of variants contained *M*=8,048,268 sites.

### Genetic relatedness inference

We computed pairwise kinship coefficients to determine family relationships using King 2.2.2 [32]. We performed inference on the set of genotype data passing quality control (see “Basic genotype quality control”) for a total of *N*=36,736 individuals and *M*=667,191 SNPs. We identified a set of unrelated individuals (*N*=35,761) up to degree 2 where individuals with kinship coefficient <0.0884 were included (“king --unrelated --degree 2”). This level of relatedness is expected since members of the same family will often be within the same healthcare system.

### Continental genetic inferred ancestry

We estimated GIA membership using a 2-step clustering procedure. First, we performed principal component analysis (PCA) [37] on all individuals in ATLAS (*N*=36,736) and samples from 1000 Genomes. Specifically, we first filtered genotypes from ATLAS by Mendel error rate (“plink --me 1 1 –set-me-missing”), founders (“--filter-founders”), minor allele frequency (“–maf 0.15”), genotype missing call rate (“--geno 0.05”), and Hardy-Weinberg equilibrium test *p*-value (“–hwe 0.001”). The filtered genotypes from ATLAS are then merged with the 1000 Genomes phase 3 dataset [28]. We align reference alleles between the two sets of data and filter out SNPs that are not an A, C, T, or G allele. Next, a 2-step LD pruning is performed on the merged dataset: (1) “--indep 200 5 1.15”, (2) “--indep-pairwise 100 5 0.1.” All filtering steps and LD pruning were performed using PLINK 1.9 [38]. This resulted in a total of 22,589 SNPs across 36,736 individuals in ATLAS. We computed the first 10 principal components using the FlashPCA 2.0 software [39] with all default settings.

For the second step, we perform clustering on the principal components to estimate GIA cluster membership for each individual in ATLAS. We use the K-nearest neighbors (KNN) algorithm where we use the superpopulation name of the samples in 1000 Genomes to define the cluster labels. The superpopulations form 5 clusters: European, African, Admixed American, East Asian, and South Asian genetic ancestry. For each ancestry cluster, we run KNN on the pair of PCs that capture the most variation for each genetic ancestry group: European, East Asian, and African ancestry groups utilize PCs 1 and 2, the Admixed American group use PCs 2 and 3, and the South Asian group use PCs 4 and 5. For each ancestry group inference, we run KNN separately. In each analysis, we use 10-fold cross-validation to select the “*k*” hyper-parameter from *k*=5, 10, 15, 20. If a sample from ATLAS had >0.50 cluster membership, then the sample is reported as the genetic inferred ancestry represented in that cluster (European genetic ancestry (GA) → European GIA, African GA → African American GIA, Admixed American GA → Hispanic Latino American GIA, East Asian GA → East Asian American GIA, South Asian GA → South Asian American GIA). See “Notes on terminology and naming conventions” for a more in-depth discussion about the naming of GIA clusters. Individuals who did not attain >0.50 membership in any cluster or were matched to multiple clusters were reported as being ‘Ambiguous GIA’. A comparison between the GIA clusters and the genetic ancestry clusters from 1000 Genomes in PC-space is visualized in Additional file 1: Fig. S2.

### Subcontinental genetic inferred ancestry

We estimate subcontinental GIA membership for individuals within the East Asian American GIA group using a 2-step clustering procedure similar to the continental GIA clustering discussed in a prior section (“Continental genetic inferred ancestry”). First, we perform PCA on all individuals in the EAA GIA group in ATLAS (*N*=3,331) and samples from the East Asian ancestry population in 1000 Genomes. Using only the genotyped SNPs, we perform the same filtering steps as described above, namely filtering ATLAS genotypes by Mendel error rate, founders, MAF > 0.15, genotype missing call rate, Hardy-Weinberg equilibrium test, and LD pruning. Following sample- and variant-level quality control, *M*=36,504 SNPs remained. We also found that not restricting to only unrelated individuals does not bias our estimates (Additional file 8: Table S16). We then compute the first 10 principal components using FlashPCA with all default settings.

For the second step, we perform clustering on the principal components to estimate subcontinental GIA cluster membership for each individual in the East Asian American GIA group in ATLAS. We use the *K*-nearest neighbors (KNN) algorithm where we use the population name of the East Asian ancestry samples in 1000 Genomes to define the cluster labels. The populations form 5 clusters: Han Chinese, Southern Han Chinese, Dai Chinese, Japanese, Kinh Vietnamese genetic ancestry. We run KNN using PCs 1–4 with 10-fold cross-validation to select the “*k*” hyper-parameter from *k*=5, 10, 15, 20. If a sample from ATLAS had >0.90 cluster membership, then the sample is reported as the genetic inferred ancestry represented in that cluster. Individuals who did not attain >0.90 membership in any cluster were reported as being “Ambiguous EAA.” A visualization of the inferred GIA clusters is visualized in Additional file 1: Fig. S3A.

Alternatively, we can define GIA clusters using self-identified information from the samples in ATLAS. We perform a similar approach as above, except we use ATLAS individuals’ self-identified race as the labels to define the clusters. We limit cluster definitions to self-identified race groups with *N*>20 for a total of 7 clusters: Chinese, Filipino, Japanese, Korean, Taiwanese, Thai, and Vietnamese. Although we do not utilize label information from 1000 Genomes, we still use the PCs computed on the merged ATLAS and 1000 Genomes dataset to keep PCA projections consistent across the 1000 Genomes-based and self-identified race-based clustering methods. We run KNN using the same procedure and thresholds as above. Again, individuals who did not attain >0.90 membership in any cluster were reported as being “Ambiguous EAA.” A visualization of the race-based inferred GIA clusters is visualized in Additional file 1: Fig. S3B. Explicit clusters could not be confidently computed for other continental GIA groups.

### IBD calling

For identity-by-descent (IBD) calling, an interim version of the ATLAS data consisting of 24,318 individuals was used. First, ATLAS data was merged with the 1000 Genome Project [28], the Simons Genome Diversity Project [40, 41], and the Human Genome Diversity Project [42, 43]. Data was filtered to remove duplicated sites and individuals or sites with more than 1% missingness. Hardy-Weinberg equilibrium was calculated in the largest SIRE groups (NH-White, HL-Oth, NH-AfAm, NH-Asian) and we removed sites that did not pass a filter of *p*-value < 1×10^−10^. We also removed individuals whose EHR sex did not match PLINK estimated genotyped sex and those who had excess heterozygosity. Lastly, we used only sites with a MAF greater than 5%. In total, 418,195 SNPs were kept for IBD analysis. The merged dataset was then statistically phased using Shapeit4 [44]. IBD was called using iLASH using default parameters [2]. For downstream analysis, IBD segments were summed between individuals to create a list of edges, where each row represented a pair of individuals, and each column represented the total genome-wide IBD between those two individuals. This matrix was filtered to remove rows representing individuals who were third degree relatives or closer, calculated using KING. We then created an undirected weighted graph using the R Package iGraph [45] where the nodes were the individuals, and the edges represented the amount of IBD shared between a pair of individuals. The InfoMap community detection algorithm, implemented in iGraph, was used to detect IBD communities [46]. InfoMap was run with default parameters. iGraph was again used for cluster visualization. The 20 largest communities were selected for visualization, and outlier nodes with degree less than 30 were removed. The graph was then visualized with the Fruchterman and Reingold force directed layout, run with 1000 iterations [47]. Each community was assigned a unique color to ease visualization.

### Genetic admixture analysis

We inferred the proportion of genetic ancestry using the ADMIXTURE software [48] under the unsupervised clustering mode with the number of clusters *k*=4, 5, 6. Specifically, we restrict to only SNPs with only an A, C, G, T allele and with MAF > 0.05 (“--maf 0.05 --snps-only ‘just-acgt’”) within ATLAS. We then merge the data from ATLAS with the 1000 Genomes phase 3 dataset and limit inference to only the subset of the overlapping SNPs. We then perform LD pruning every 2 kb on the merged dataset (“--bp-space 2000”). All filtering steps and LD pruning were performed using PLINK. This resulted in a total of 223,095 SNPs across 36,736 individuals in ATLAS which was then used for ancestry inference using ADMIXTURE. We also found that not restricting to only unrelated individuals does not bias our estimates (Additional file 8: Table S16).

Finally, we performed the admixture analysis with “./admixture atlas_1kg_bed_fille k” with *k* equal to 4, 5, or 6. We compare the ancestry proportions from each SIRE to estimate the ancestry represented in each mixture component. For *k*=4, we label the component with the majority of NH-White individuals as European ancestry, the component with the majority of NH-AfAm individuals as African ancestry, the component with the majority of NH-Asian individuals as East Asian ancestry, and the component with the highest number of HL-Other and HL-White individuals as Native American ancestry.

### Phecodes

Billing codes documented in the medical record were used to generate phenotypes for analysis. The previously described *phecode* ontology (v1.2) maps the specific ICD-9 and ICD-10 codes from each patient’s chart onto a group of >1800 more general and clinically meaningful phenotype terms [49]. Mapping completed with the PheWAS R package [50] (https://github.com/PheWAS/PheWAS) creates binary phenotypes. Patients with one or more instances of a phecode were considered cases while patients without any instance of the corresponding phecode were considered controls. We limited analyses to phecodes with at least N-Case > 50 in each GIA group for a total of 1568 phecodes meeting this threshold in the EA GIA, 802 in the AA GIA, 1223 in the HL GIA, and 891 in the EAA GIA group.

### Role of phecode occurrences for defining cases

We define a phecode occurrence as an encounter containing at least one of the ICD codes specified in the phecode definition. If a corresponding ICD code is found on another separate encounter, we treat this instance as a separate phecode occurrence. We compare two definitions of phecodes. For the first definition, we only require the presence of an ICD code attached to any type of patient encounter (i.e., laboratory tests, hospital, outpatient, medications, telehealth appointments, notes, phone calls). For the second definition, we require the presence of an ICD code attached to only encounters from appointment, office, hospital, or procedure visits. This stricter definition attempts to avoid capturing encounters that may be less indicative of a diagnosis (e.g., patient-physician telehealth messaging). We refer to these two definitions as all-encounter-derived and visit-derived phecodes. Using these two types of definitions, we then vary the number of phecode occurrences required for defining cases and compute the proportion of retained cases compared to the sample sizes if only 1 occurrence was required.

### Association between phecodes and genetic ancestry

To test the differential prevalence of phecodes across genetic ancestry group, we performed a marginal association test for each phecode to compare its prevalence in one of the genetic ancestry groups (EA, AA, HL, and EAA) with the other three groups using the following logistic regression model:

$$\mathrm{logit}\left(\mathrm{phecode}\right)={\beta}_0+{\beta}_1\mathrm{genetic}\_\mathrm{ancestry}\_\mathrm{group}+{\beta}_2\mathrm{sex}+{\beta}_3\mathrm{age}\ \left[\mathrm{over}\ \mathrm{all}\ \mathrm{ATLAS}\ \mathrm{individuals}\right]$$

To account for the potential confounding effects of SIRE, we performed additional analyses with the model:

$$\mathrm{logit}\left(\mathrm{phecode}\right)={\beta}_0+{\beta}_1\mathrm{genetic}\_\mathrm{ancestry}\_\mathrm{group}+{\beta}_2\mathrm{sex}+{\beta}_3\mathrm{age}+{\beta}_4\mathrm{SIRE}\ \left[\mathrm{over}\ \mathrm{all}\ \mathrm{ATLAS}\ \mathrm{individuals}\right]$$

Statistical significance was determined after correcting for the number of tested phecodes with Bonferroni correction procedure (*p*-value<1.12×10^−05^).

We also applied the method to the East Asian American group to test the phecode prevalence difference across subcontinental ancestry groups including Chinese, Japanese, Filipino, and Korean Americans.

### Association between genetic admixture proportions and phecodes

Given the substantial variation of admixture proportion within each SIRE group, we test the association of phecode with admixture proportion (*k*=4) for 600 phecodes within each of the seven ATLAS SIRE groups (NH-White, NH-AfAm, HL-Other, HL-White, NH-Asian, NH-PI, NH-AmIn) with the following model:

$$\mathrm{logit}\left(\mathrm{phecode}\right)={\beta}_0+{\beta}_1\mathrm{admixture}\_\mathrm{proportion}+{\beta}_2\mathrm{sex}+{\beta}_3\mathrm{age}\ \left[\mathrm{over}\ \mathrm{individuals}\ \mathrm{within}\ \mathrm{a}\ \mathrm{SIRE}\right]$$

Each model is limited to individuals of one SIRE instead of all ATLAS individuals. Only traits with >10 cases per SIRE were tested. Significance is determined after adjusting for the number of tested phenotypes with Bonferroni correction procedure (*p*-value <2.08×10^−05^).

### GWAS quality control per GIA

When performing GWAS, we stratified individuals by GIA groups and then performed an additional level of QC separately within each GIA group. We limited analyses to the 4 largest GIA groups: European American (*N*=22,380), African American (*N*=1995), Hispanic Latino American (*N*=6073), and East Asian American GIA (*N*=3331). At this time, we omitted GWAS analyses within the South Asian American GIA group due to the limited sample size (*N*=625). Individuals who could not be clustered into a specific GIA group (*N*=2332) were also omitted from GWAS analyses.

For GWAS, we utilized imputed data consisting of 8,048,268 SNPs across *N*=36,736 individuals. Within each ancestry group, samples identified as heterozygosity outliers (+/− 3 SDs from the mean) were removed and SNPs that failed the Hardy-Weinberg equilibrium test (*p*-value <1×10^−12^) were also removed. Finally, we limited analyses to only SNPs with MAF > 1% within each GIA group, yielding a total of *N*=22,380 individuals and *M*=6.0 million SNPs within the European American GIA group, *N*=1995 individuals and *M*=5.9 million SNPs within the African American group, *N*=6073 and *M*=6.3 million SNPs within the Hispanic Latino American group, and *N*=3331 individuals and *M*=4.8 million SNPs within the East Asian American group.

### Ancestry-specific GWAS

GWAS for all 6 traits were performed separately within each of the 4 continental ancestry groups that met the minimum *N*>50 cases. Additional GWAS-specific quality control is performed within each GIA group (see GWAS quality control per GIA). Using marginal logistic regression implemented in PLINK, we computed association statistics at each imputed autosomal SNP (“plink --logistic beta”). We additionally used age, sex, and PCs 1–10 as covariates where age is defined as the individuals’ current age within the EHR (as of September 2021). The values used to represent sex in this specific analysis are derived from patients’ self-identified sex as reported in the EHR. Within the EHR, this specific field is labeled as “Sex” and has a list of pre-determined multiple-choice fields where participants select one of the following options: “Male,” “Female,” “Other,” “Unknown,” “*Unspecified,” “X.” We find that 45.1% of individuals self-identify as male and 54.9% self-identify as female.

### Meta-analyses

We perform meta-analyses for each trait across all GIA groups. First, we run ancestry-specific GWAS (see “Ancestry-specific GWAS”) within each GIA group with adequate sample size (*N*-Cases>50). We exclude analyses where very few of the SNPs produced a valid (non-NA) *p*-value which is likely attributed to a small sample size. The meta-analysis for skin cancer consisted of measurements from the EA and HL GIA groups; EA, AA, HL, and EAA GIA groups for ischemic heart disease; EA, AA, HL, and EAA GIA groups for chronic nonalcoholic liver disease; AA and HL GIA groups for uterine leiomyoma; HL and EAA GIA groups for liver/intrahepatic bile duct cancer; EA, AA, and HL GIA groups for chronic kidney disease. We performed each meta-analysis using a fixed effect model as implemented in PLINK (“--meta-analysis + logscale”). Association statistics computed from the meta-analyses are reported for SNPs that occur in at least two of the GIA groups.

### PheWAS

We perform a PheWAS on the top SNPs from each ancestry-specific GWAS analysis that met genome-wide significance (*p*-value <5×10^−8^). Only phecodes with at least *N*-Cases>50 per GIA group were considered, resulting in a total of 1568 phecodes meeting this threshold in the EA GIA and 1223 in the HL GIA. Analyses in the AA and EAA GIA groups were excluded since the top SNPs were not significantly associated in these groups. We additionally stratified individuals by sex for the sex-specific phecodes, which are denoted in the definition of each phecode. This resulted in a total of 24 male- and 113 female-specific phecodes within the EA GIA group, and 12 male- and 87 female-specific phecodes within the HL group after limiting to phecodes with at least *N*-Cases > 50. We used individuals’ self-identified sex as reported in the EHR for this analysis.

We performed an association test between the top SNP and all phecodes in a given GIA group under a logistic regression model. Age, sex, and PCs 1-10 were used as covariates in the regression where age is defined as the individuals’ current age within the EHR (as of September 2021), and sex is derived from individuals’ EHR. The association test is performed using the logistic regression option implemented in PLINK (“plink --logistic beta”). The PCs used in the regression analysis were re-computed using only on individuals from within each respective GIA group. Phenotype significance was determined as *p*-value <0.05/(# phecodes), thus each GIA group has a specific significance threshold due to the different number of tested phecodes. A more stringent threshold also accounting for genome-wide significance is also computed where *p-*value <5×10^−8^/(# phecodes). Both thresholds are denoted in the PheWAS plots.

### Effective sample size of associated phecodes

To assess the power of the PheWas analysis at rs2294915 between the European American (EA) GIA and Hispanic Latino American (HL) GIA groups, we compute the effective sample size (*N*_eff_) of each associated phecode, where the effective sample size balances the number of cases and controls when measuring the power of an analysis [51]: *N*_eff_ *=* 2 / (1/*N*_cases_ + 1/*N*_controls_*).*

## Results

### ATLAS includes individuals of diverse continental ancestries

The UCLA Health patient population is diverse, with 65.36% self-identifying their race as White, 5.23% as Black or African American, 9.89% as Asian, 0.41% as Native American or Alaska Native, 0.31% as Pacific Islander, and 18.81% identify as one of the additional races listed in detail in the Additional file 2 (Table S1, S3). For ethnicity, a separate concept from race and recorded under a different field in the EHR, 15.96% of individuals self-identify as Hispanic or Latino; the remaining individuals self-identify as non-Hispanic/Latino (Additional file 2: Table S2, S3). We define genetic ancestry as the characterization of the population(s) from which an individual is biologically descended and the genetic relationship between an individual and these ancestors. When information describing the origin of individuals’ recent biological ancestors is not available, we can instead infer the genetic ancestry using statistical methods. We introduce the term “genetically inferred ancestry (GIA)” to describe the genetic characterization of individuals within a group who likely share recent biological ancestors as inferred by a method of choice. We emphasize that GIA differs from genetic ancestry in that GIA is highly dependent on the inference method (e.g., PCA, IBD) and choice of reference data. We provide a discussion about the rationale behind the terminology and naming conventions used in this work under the section “Notes on terminology and naming conventions”.

**Fig. 1:**
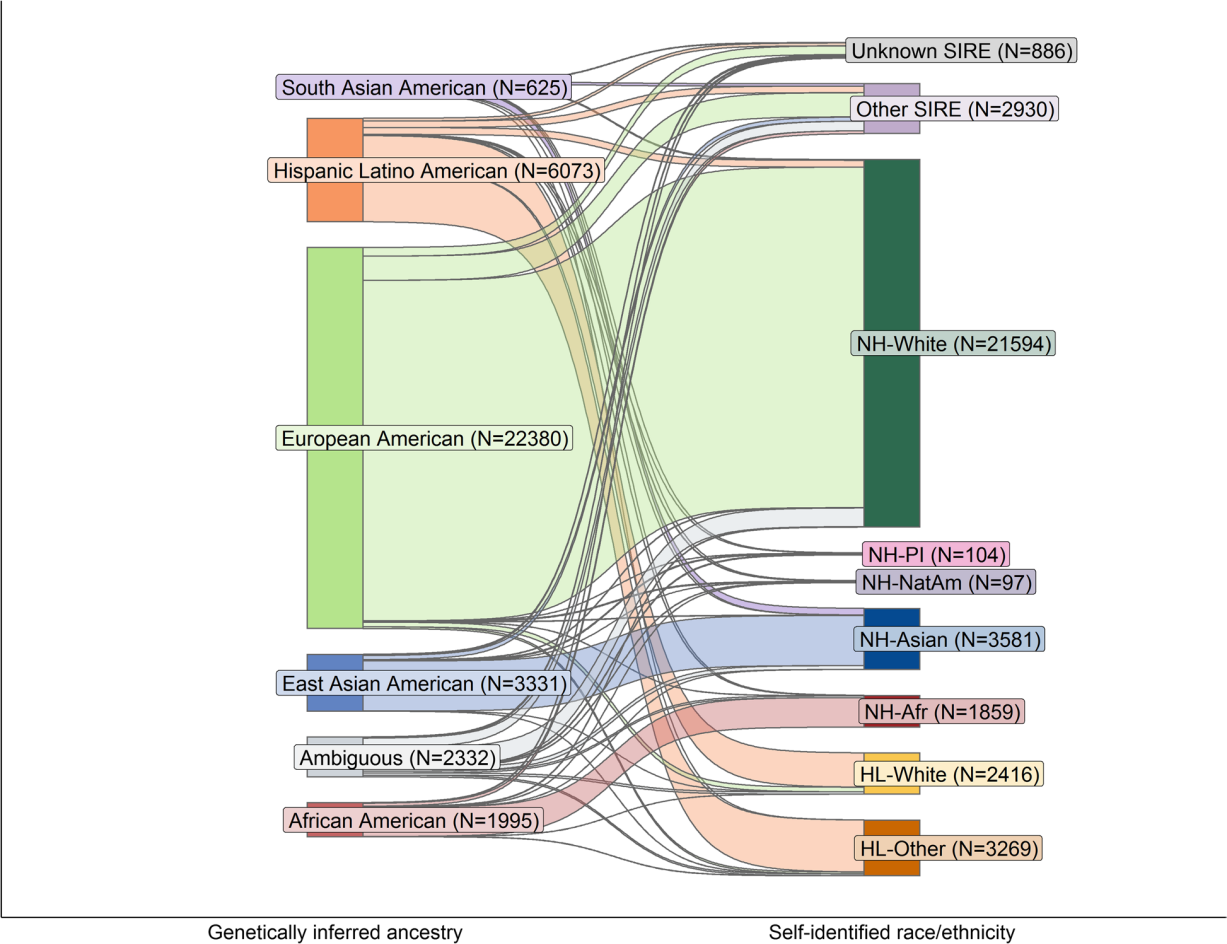
Self-identified race/ethnicity (SIRE) and genetically inferred ancestry (GIA) are not analogous. We show a Sankey diagram visualizing the sample size breakdown of individuals in each genetically inferred ancestry group and SIRE groups for all individuals in ATLAS (*N* = 36,736)

Using data from the 1000 Genomes Project [28], we investigated genetically inferred ancestry in ATLAS through principal component analysis (PCA) [37, 52] and clustering techniques (see “Methods”). Using the five continental ancestry populations within 1000 Genomes (European, African, Admixed American, East Asian, South Asian ancestry) as reference, we identify clusters of individuals with European American, African American, Hispanic Latino American, East Asian American, and South Asian American genetically inferred ancestry (Additional file 2: Table S4, Additional file 1: Fig. S1, S2). Although we broadly find that self-identified race and ethnicity highly correlates with an individual’s inferred genetic ancestry, we still observe marked differences between the two (Fig. 1). For example, we find 10.63% of individuals within the European American GIA cluster do not identify as being within the Non-Hispanic/Latino – White (NH-White) SIRE; 13.33% of individuals within the African American GIA cluster do not self-identify as Non-Hispanic/Latino – Black/African American (NH-AfAm), and 16.58% of the Hispanic Latino American cluster do not identify as Hispanic/Latino – Other Race (HL-Other) or Hispanic/Latino – White (HL-White) (Additional file 2: Table S5). This further emphasizes that SIRE is not equivalent to GIA and that these two concepts form distinct groupings.

**Fig. 2:**
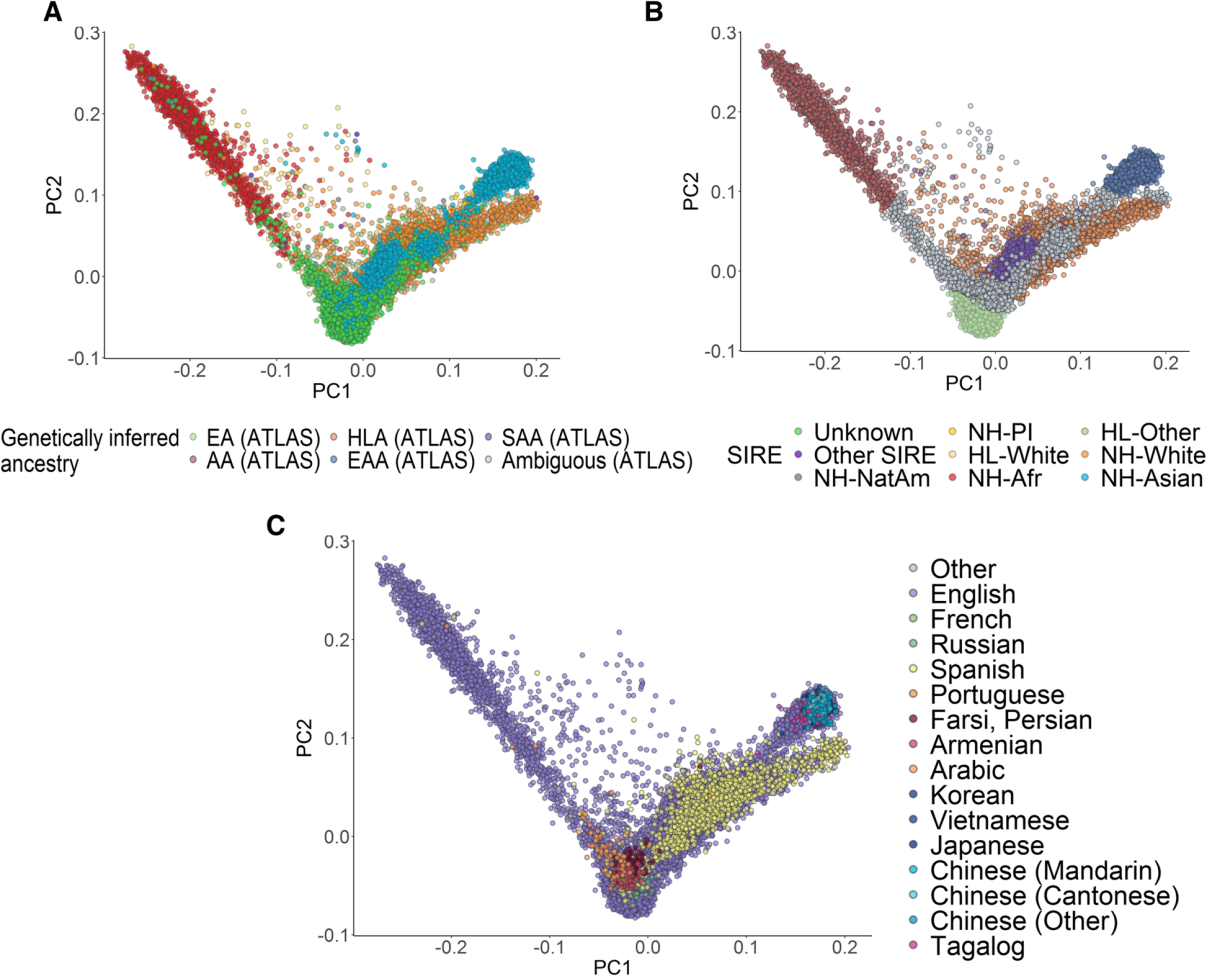
Global PCA reflects self-identified race/ethnicity and language of ATLAS participants. **A** Genetic PCs 1 and 2 of individuals in ATLAS (*N*=36,736) shaded by continental GIA as inferred from 1000 Genomes. **B, C** The first two genetic PCs of the ATLAS participants shaded by SIRE and preferred language, respectively. To improve visualization in **C**, only languages with >10 responses were assigned a color

Further emphasizing the distinction between GIA and SIRE, we reveal extensive genetic heterogeneity both between and within SIREs, as observed from the orthogonal spectra from PCA (Fig. 2A, B). For example, most individuals who self-report as NH-AfAm lie along a cline between the AA and EA GIA clusters. However, 102 individuals in this SIRE cannot be clustered into either the AA or EA ancestry cluster. This is likely because many of these individuals in ATLAS self-identify as African American, which suggests genetic admixture between African and European ancestry in this group. We also find that the NH-Asian SIRE has individuals spread along all PC1 and PC2, spanning the entire space between the EAA and EA GIA clusters (Fig. 2B). However, when looking solely at GIA, we are not able to observe this pattern. Instead, most individuals in between these two clusters were inferred to have ambiguous GIA, where specifically, 221 individuals within the NH-Asian SIRE were not able to be clustered into a specific GIA group. Overall, 6.35% of individuals still have unclassifiable genetic ancestry (Additional file 2: Table S4) either because they were clustered into multiple GIA groups or none at all. The latter could be due to extensive admixture in their genomes or the absence of relevant ancestral groups in the chosen reference panels.

Categorizing individuals by self-identified preferred language, we observe trends that are consistent with both SIRE and continental GIA (Fig. 2C). For example, out of all individuals who report Spanish as their preferred language, 94.47% of these individuals were estimated to have Hispanic Latino American GIA. Additionally, 99.76% of individuals who report Japanese, Korean, Tagalog, Vietnamese, Mandarin Chinese, or Cantonese as their primary languages were inferred to have East Asian American GIA. We also observe clusters of individuals who speak Armenian, Arabic, and Farsi/Persian; we find that 47.13% of the individuals that speak these languages could not be classified into one of the five continental GIA groups. This discrepancy is likely because the 1000 Genomes reference panel does not contain samples from regions where these languages are primarily spoken. These findings showcase the limitation of current reference panels of genetic diversity and demonstrate the value of characterizing individuals using both genetic ancestry and self-identified information.

### Fine-scale subcontinental ancestry within ATLAS individuals

**Fig. 3:**
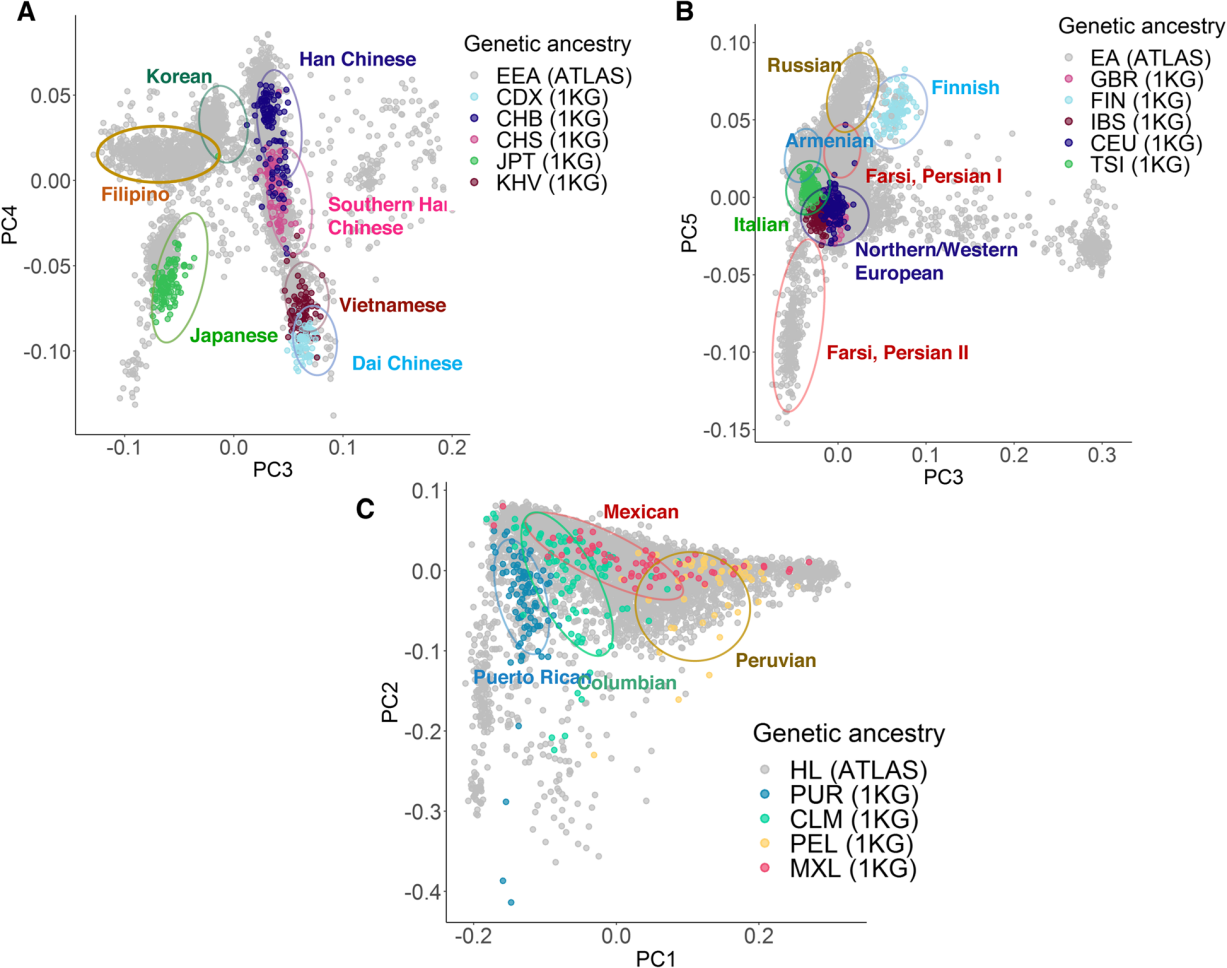
PCA of individuals with inferred East Asian American, European American, and Hispanic Latino American genetic ancestry in ATLAS captures fine-scale subcontinental ancestry groupings. PCA was performed separately within each continental GIA in ATLAS with the corresponding subcontinental ancestry samples from 1000 Genomes: **A** East Asian American, **B** European American, **C** Hispanic Latino American. Cluster annotation labels were determined using a combination of samples from 1000 Genomes and self-identified race, ethnicity, and language information from the EHR

Next, we assessed genetic ancestry at the subcontinental level. Performing PCA only on individuals from the EAA GIA group from ATLAS and the East Asian ancestry group from 1000 Genomes, we observe distinct clusters of individuals as shown in Fig. 3A, where the cluster annotations provide a visual reference describing the approximate location and size of GIA clusters (as opposed to the formal cluster membership thresholds). Shading by the subcontinental East Asian genetic ancestry groups present in 1000 Genomes, we observe clusters corresponding to three different subgroups of Chinese ancestry (Han Chinese, Southern Han Chinese, and Dai Chinese). Additionally, we see clusters of both Japanese and Vietnamese ancestry. Using 1000 Genomes as a reference panel, we can use a *K*-nearest neighbors clustering approach to infer the subcontinental genetic ancestry of individuals in ATLAS where we find *N*=307 in the Han Chinese American GIA cluster, *N*=224 in the Southern Han Chinese American GIA cluster, *N*=483 in the Japanese American GIA cluster, and *N*=136 in the Vietnamese American GIA cluster (see “Methods”; Additional file 1: Fig. S4A). There were not any ATLAS individuals assigned to the Dai Chinese American GIA cluster. When projecting ATLAS individuals’ preferred language onto the PCs, two distinct clusters are delineated according to the Chinese Mandarin and Chinese Cantonese/Toishanese languages (Additional file 1: Fig. S3B). The Southern Han Chinese American cluster of individuals correlates with individuals speaking Chinese Cantonese/Toishanese, where 37.50% of individuals who speak Chinese Cantonese/Toishanese are within this cluster. The Han Chinese American cluster correlates with Chinese Mandarin where 45.33% of individuals who speak Chinese Mandarin fall within this cluster.

From Fig. 3A, there are two notable clusters that do not match any of the East Asian subcontinental ancestries represented within 1000 Genomes. Projecting ATLAS individuals’ self-identified preferred languages onto the PCs shows that many of these individuals in these two clusters self-identify as speaking Korean and Tagalog (Additional file 1: Fig. S3B). These patterns are similarly reflected by individuals’ self-identified race where the majority of these individuals self-identify as Korean and Filipino (Additional file 1: Fig. S3A). Because there is descriptive self-identified demographic information available in the EHR, we can alternatively use this to define subcontinental GIA clusters in ATLAS. This could be advantageous since a >65.48% (*N*=2181) ATLAS individuals within the EA GIA group could not be further clustered into a subcontinental GIA group derived from 1000 Genomes. Using self-identified race groups with *N*>20 individuals, we repeat the same clustering process described above using individuals’ self-identified race as cluster category labels. Using self-identified race information over information available in 1000 Genomes, we are able to recover two large clusters consisting of individuals with Korean American (*N*=533) and Filipino American (*N*=761) GIA as well as identify additional clusters of individuals corresponding to Thai (*N*=33) and Taiwanese (*N*=73) GIA (Additional file 1: Fig. S4B, Table S4). This clustering not only characterizes the fine-scale genetic and ethnic diversity of ATLAS, but also emphasizes how self-reported information such as primary spoken language can be combined with genetic information to identify patterns not otherwise evident.

Next, we looked at individuals with subcontinental genetic ancestry of European descent in ATLAS, but due to limitations in the 1000 Genomes reference panel, we were unable to describe the origins of the majority of the observed genetic variation within the ATLAS European American GIA cluster (Fig. 3B). Comparing self-identified race and ethnicity information also did not delineate any subgroups since most individuals fell within the NH-White SIRE (Additional file 1: Fig. S5A). Instead, we project individuals’ preferred language onto the projected PCs. Aside from English, we observe clusters of individuals whose preferred languages are Arabic, Armenian, and Farsi/Persian. Notably the primary populations that speak these languages are not present in the current 1000 Genomes reference panel (Additional file 1: Fig. S5B). Although not a definitive determination of ancestral origin, these results suggest that individuals in these clusters may have cultural ties relating to the Middle East. We also observe two distinct clusters of individuals who speak Farsi/Persian (labeled as “Farsi, Persian I” and “Farsi, Persian II” in Fig. 3B), suggesting that although these groups may share cultural and/or ethnic ties, the groups could have multiple ancestral origins. However, due to limited genetic and self-identified information, we did not attempt to formally infer the subcontinental ancestry for these individuals.

We perform a similar analysis for the Hispanic Latino American cluster of individuals where we re-ran PCA only on individuals in the HL GIA cluster within ATLAS and individuals from the Admixed American population in 1000 Genomes. Projecting population labels from 1000 Genomes onto the PCs, we observe relatively sparse clusters of individuals of Mexican, Peruvian, Colombian, and Puerto Rican ancestry from 1000 Genomes (Fig. 3C). Due to the overlapping and sparse shape of these clusters, we did not attempt to formally infer subcontinental ancestry for these individuals. Overlaying SIRE and language as previously discussed also did not reveal any additional population structure in this group (Additional file 1: Fig. S6). Since the HL GIA group is inherently an admixed population, we instead shade the PCs by the estimated proportions of European and Native American ancestry (see “Methods”; Additional file 1: Fig. S6B, C). We observe a cline between European and Native American ancestries, demonstrating that although we cannot determine discrete clusters within our data, there is still substantial population structure present.

Corresponding analyses were also performed for the African American GIA group in ATLAS, but clear subcontinental clusters could not be constructed from reference panel information (Additional file 1: Fig. S7A). Similarly, SIRE information did not delineate any clusters nor did preferred language (Additional file 1: Fig. S7B, C). Since the majority of patients self-identify as African American, an admixed population of African and European ancestry, we project the proportion of European and African ancestry onto the PCs (Additional file 1: Fig. S7D, E). We observe a cline going from higher proportions of European ancestry to higher proportions of African ancestry. This suggests that for very admixed populations, it would be more advantageous to quantify population substructure continuously rather than within discrete categories. We omitted the subcontinental analysis for the South Asian American GIA group due to the small sample size (*N*=625).

### IBD sharing reveals communities of recent shared ancestry within ATLAS

**Fig. 4:**
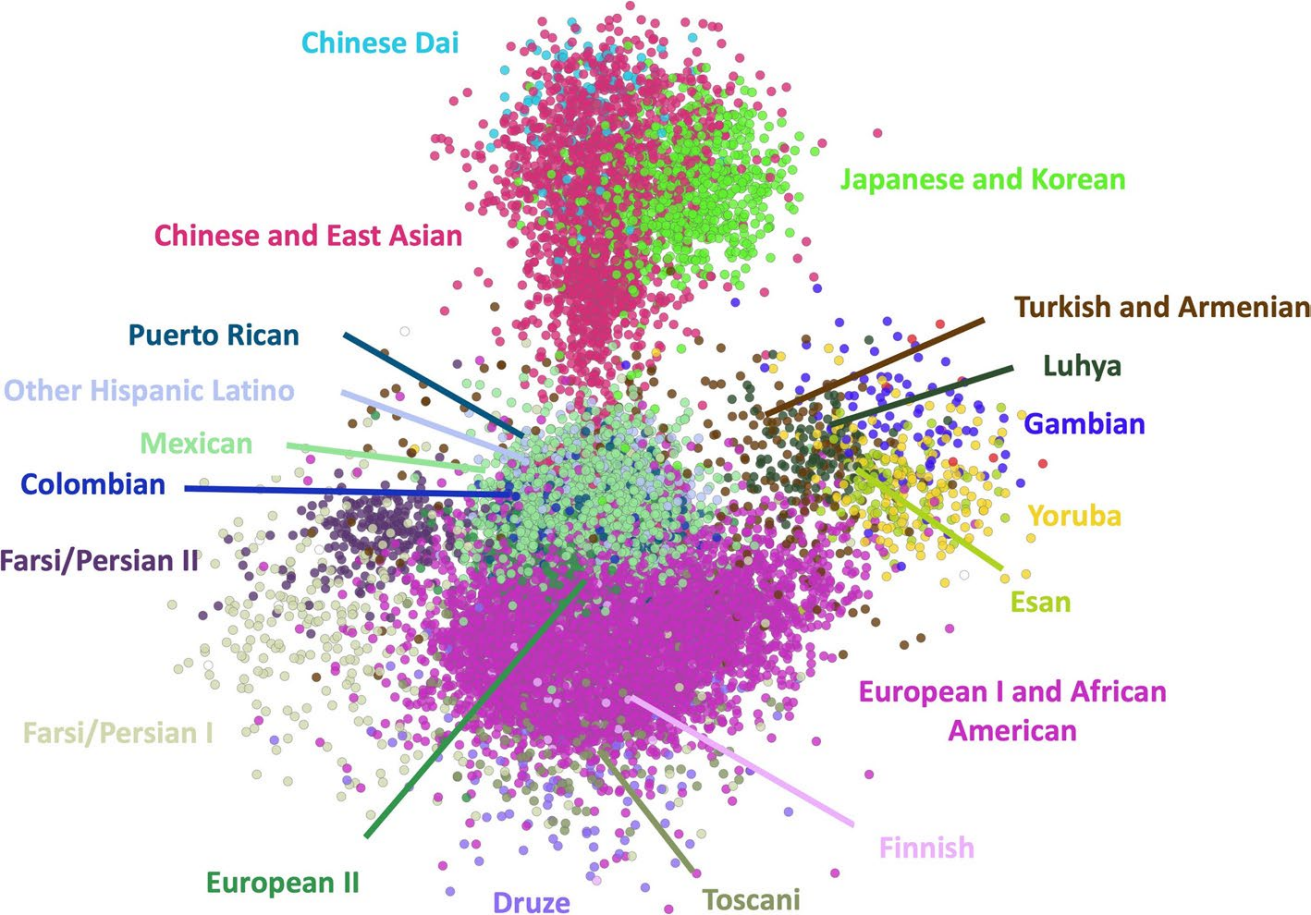
IBD sharing between ATLAS participants. InfoMap community membership is indicated by color for all communities with >100 individuals (20 communities total) and individuals with a degree >30. Community membership indicates elevated shared IBD within that community. Community identity is labeled adjacent to the network plot in the corresponding color

A complementary method to principal components for inferring fine-scale ancestry is identical-by-descent (IBD) analysis [53–55]. Using pairwise IBD estimates for all individuals in ATLAS and reference population information from the 1000 Genomes Project [28], Simons Genome Diversity Project [40], and Human Genome Diversity Project [42], we describe fine-scale populations based on total pairwise IBD (Fig. 4; see “Methods”). Each subgroup is annotated according to a combination of genetic ancestry from reference populations as well as self-identified race, ethnicity, and language information. Many subgroups have similar characteristics to those defined from PCA-based methods, such as the Filipino and Dai Chinese clusters. We can also characterize subgroups not previously identified through the previous PCA analysis. For example, PCA-based methods were only able to distinguish clusters at the level of continental African ancestry, whereas IBD clustering identified West African, East African, and Ethiopian subgroups. In contrast, Japanese and Korean individuals form a single subgroup when estimated by the IBD clustering approach, whereas PCA-based methods delineated these individuals into two separate groups. Note that both IBD and PCA-based methods’ granularity is dependent on the clustering algorithm used and here we report at only a single level of resolution. For further discussion of PCA and IBD for fine-scale population analyses, see Belbin et al. [56]. These results show that each stratification method identifies distinctive features to infer fine-scale subgroups. These techniques can then be combined to divide a population into more descriptive subgroups. A more in-depth IBD analyses within ATLAS is described in additional work [57].

### Admixture describes genetic variation within self-identified race/ethnicity groups

As demonstrated in prior sections, many individuals do not fall within a single GIA cluster, but instead lie on the continuum between multiple ancestry groups. We can characterize this variation through genetic admixture, the exchange of genetic information across two or more populations [58]. We estimate genetic ancestry proportions using *k*=4, 5, or 6 ancestral populations and visualize groups of individuals by SIRE (see “Methods”; Additional file 1: Fig. S8). For the following analyses, we use *k*=4 ancestral populations where the clusters correspond to European, African, Native American, and East Asian ancestry. Among individuals in the NH-AfAm SIRE, the estimated average proportion of European ancestry is 24% and 73% African ancestry (Additional file 2: Table S6). We also observe that the HL-Other and HL-White SIREs have approximately the same admixture profile, where the proportion of European ancestry is 48% and 58% respectively, 6% and 5% African ancestry, and 44% and 35% Native American ancestry. This admixture profile is consistent with individuals of Mexican ancestry where there is mainly European and Native American ancestry [59]. However, there is also a large amount of variation within SIREs, where for example, individuals who identify as Hispanic or Latino ethnicity are estimated to have European ancestry percentages ranging from nearly 0% to almost 100%.

### Genetic ancestry groups correlate with disease prevalence within ATLAS

**Fig. 5:**
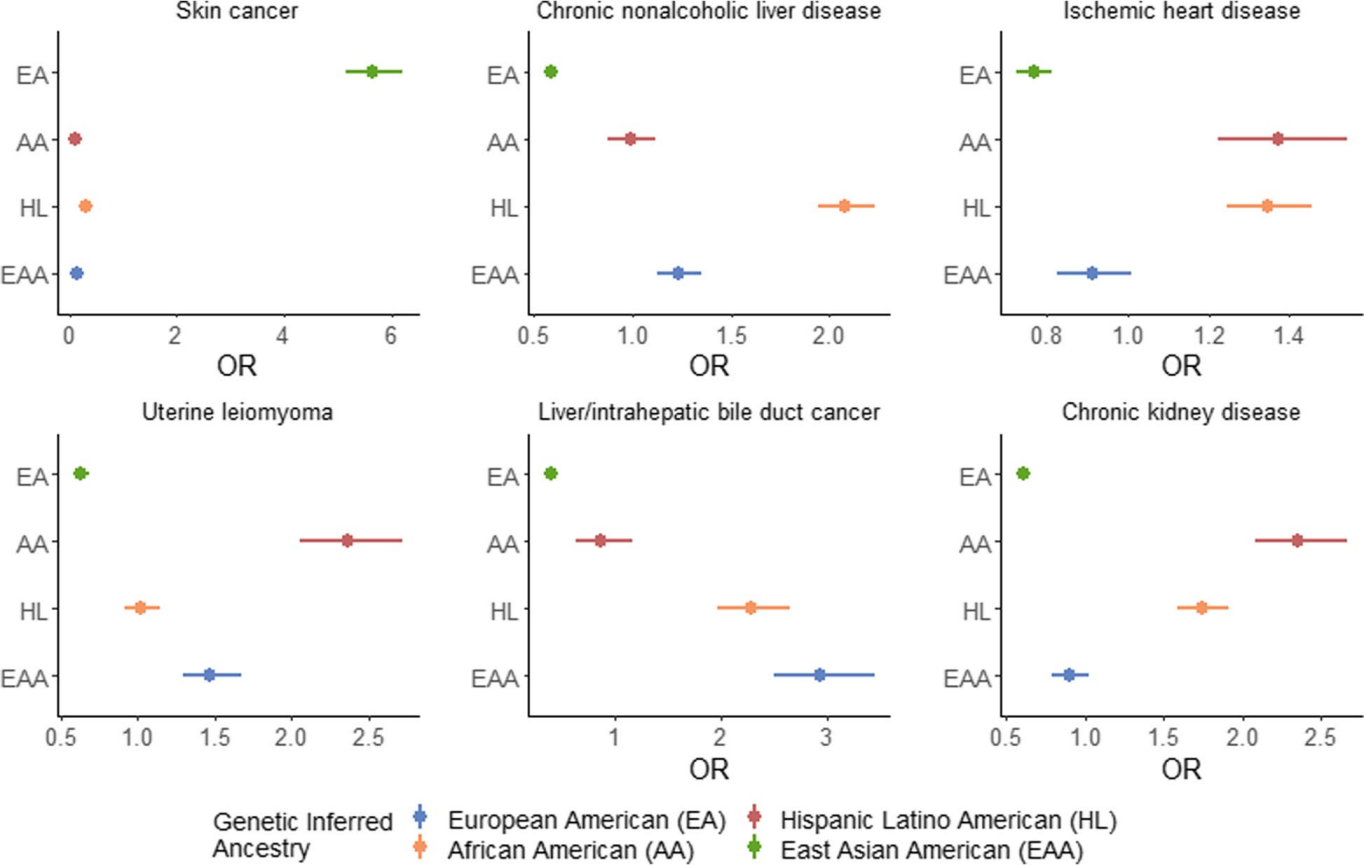
Disease associations vary across continental genetically inferred ancestry groups in ATLAS. We show the odds ratio computed from associating each phenotype with individuals’ genetically inferred ancestry in ATLAS (*N*=36,736) under a logistic regression model. Error bars represent 95% confidence intervals

Understanding how disease prevalence varies across populations is integral to understanding how the interplay of genetic factors and social determinants of health contribute to disease risk. We investigated over 1500 EHR-derived phenotypes (phecodes) [49] from across a wide set of disease groups. We define cases as individuals having the presence of at least one occurrence of the specified phecode (see “Methods”). We find that varying the number of required phecode occurrences and types of encounters when defining cases does not substantially change case and control assignment in this dataset (Additional file 1: Fig. S19, Table S13). Limiting our analyses to phecodes with a minimum of 50 cases, we identify 1512 total significant phecode-GIA associations across the 4 largest continental GIA groups after adjusting for age and sex (*p*-value<1.12×10^−5^; Bonferroni correction for all phecodes tested across 4 GIA groups) (Additional file 3: Table S7). Overall, there are 732 phenotypes that show cross-ancestry differences whose prevalence varies significantly by GIA. From this set of significant associations, the highest number of phecodes are from the circulatory (*N*=89), endocrine/metabolic (*N*=84), and digestive (*N*=90) system-related categories. Specifically, we recapitulate many known associations such as skin cancer (*p*-value=3.13×10^−281^) in the EA GIA group [36, 37]; chronic nonalcoholic liver disease in the HL GIA group (*p*-value=4.83×10^−97^); ischemic heart disease (*p*-value=6.74×10^−08^), chronic kidney disease (*p-*value=1.98×10^−41^) and uterine leiomyoma (*p*-value=2.30×10^−33^) in the AA GIA group [60–63], and liver and intrahepatic bile duct cancer (*p*-value=1.85×10^−38^) within the EAA GIA group [32, 34, 35] (Fig. 5).

To further explore the implications of genetic ancestry for a range of diseases, we focus on 6 phenotypes that were found to be significantly associated with genetically inferred ancestry (GIA) in ATLAS. This set represents a wide variety of diseases: skin cancer, ischemic heart disease, chronic nonalcoholic liver disease, uterine leiomyoma, chronic kidney disease, and liver/intrahepatic bile duct cancer. Our goal was to capture variation across each GIA group: ischemic heart disease, chronic kidney disease, and uterine leiomyoma have the strongest association with the African American GIA group, skin cancer with the European GIA, chronic nonalcoholic liver disease with the Hispanic Latino American GIA, and liver/intrahepatic bile duct cancer with the East Asian American GIA group (Additional file 3: Table S7). Additionally, previous literature has already shown that the prevalence of these 6 diseases has some level of variation across racial and ethnic groups, making them ideal candidates for the further analysis of disease variation across GIA groups in ATLAS [59–70].

The GIA clusters are often correlated with SIRE, as demonstrated in previous sections. To assess whether the observed effect is primarily driven by the role of genetic ancestry, we also add individuals’ SIRE as a covariate into the model. After multiple hypothesis testing (Bonferroni correction for all tested phecodes across 4 GIA groups: *p*-value <1.12×10^−5^), we replicate 259 out of 1512 phecode-GIA associations despite the reduced effect magnitude and association significance (Additional file 4: Table S8). Out of the 6 example traits, all but the 2 within the NH-AfAm SIRE maintained significance (Additional file 1: Fig. S9). This demonstrates that there is some level of disease association attributed to the ancestry component. Incorporation of SIRE should not be interpreted as formal adjustment for environmental factors. However, SIRE could capture sociocultural and socioeconomic factors that are not explicitly modeled and/or available to use through the EHR.

We also observe substantial disease risk heterogeneity across subgroups of the same continental GIA group. We perform association tests between subcontinental GIA and phecodes within the East Asian American GIA group in ATLAS for phenotypes with *N*>20 cases (Additional file 3: Table S7). To maximize sample size, we use the race-based GIA clusters (see “Methods”) and limit analyses to the Korean (*N*=552 individuals, 546 phenotypes), Japanese (*N*=548 individuals, 600 phenotypes), Filipino (*N*=844 individuals, 700 phenotypes), and Chinese (*N*=1217 individuals, 812 phenotypes) GIA subgroups in ATLAS. Across subgroups, we observe disease associations to varying degrees (Additional file 1: Fig. S10). We find 3 significant associations with subcontinental GIA and phenotypes where significance was determined after correcting for 812 tested phecodes, *p*-value<6.16×10^−5^ (see “Methods”). For example, the direction of the association with chronic kidney disease, varies across subcontinental GIA groups where the odds ratio for the Chinese American GIA group is 0.54 (*p*-value=2.9×10^−5^) but the odds ratio for the Filipino American GIA group is 1.83 (*p*-value=2.87×10^−5^). Additionally, the odds ratio estimated for ischemic heart disease in the Filipino American GIA subgroup is 1.81 (*p*=3.33×10^−7^), but performing the association at the continental EAA GIA level, a conclusive trend cannot be determined (OR 0.91, *p*-value=7.10×10^−2^). These results indicate that genetically grouping individuals across subcontinental GIA groups yields meaningful interpretation of disease risk across groups of individuals that might be missed when only grouping individuals at the continental level.

**Fig. 6:**
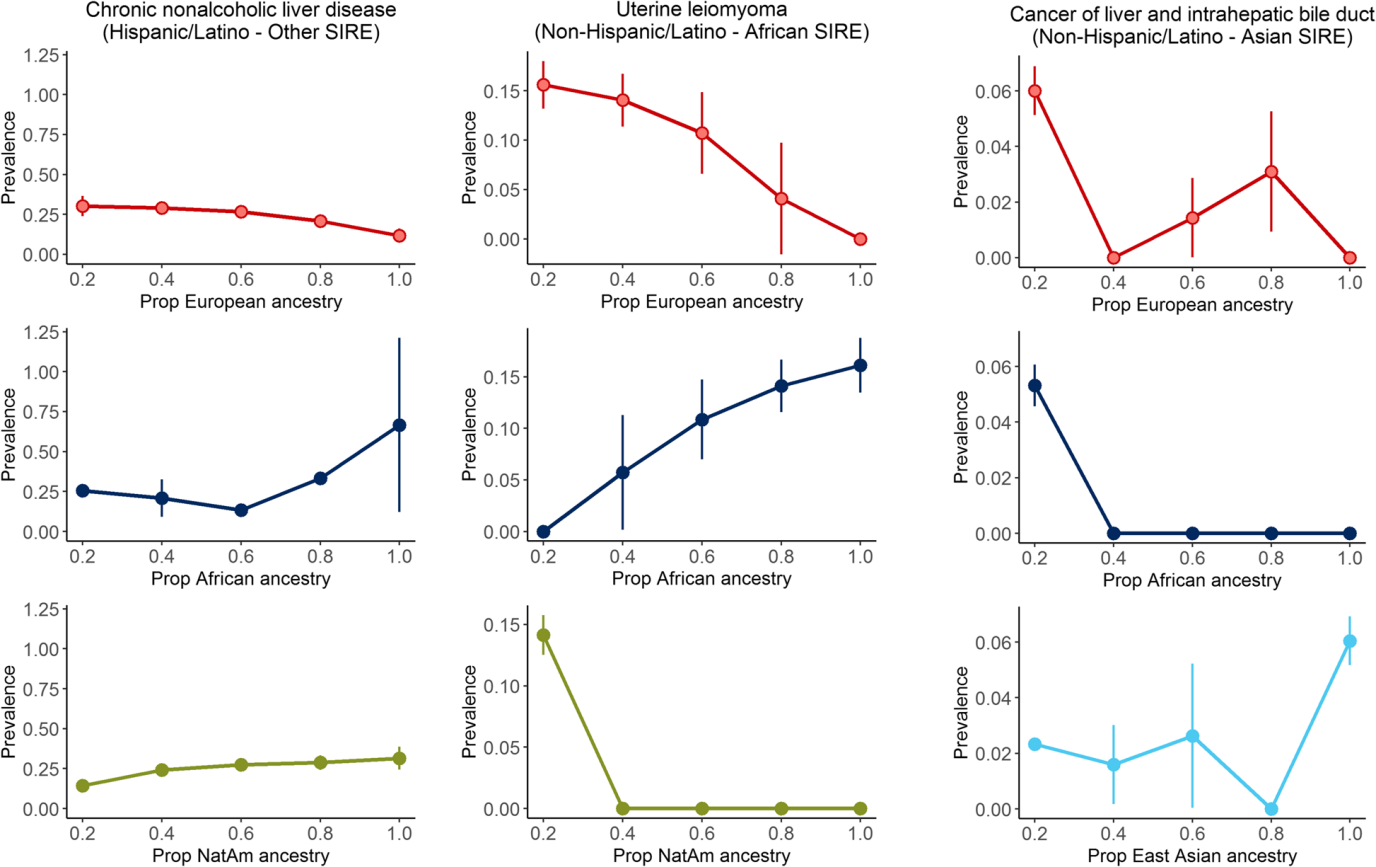
Global ancestry correlates with disease prevalence in admixed individuals. Individuals by SIRE who have had a diagnosis of **A** chronic nonalcoholic liver disease, **B** uterine leiomyoma, or **C** liver/intrahepatic bile duct cancer are binned by their proportions of either European, African, Native American, or East Asian ancestry estimated using ADMIXTURE. Within each bin, we plot the prevalence of the diagnoses and provide standard errors (+/− 1.96 SE) of the computed frequencies

We also investigated disease prevalence within admixed individuals where variation in genetic ancestry across individuals in the population allows for the correlation of disease risk with the proportion of genetic ancestry from any given continental group. Within each SIRE group, we perform an association test between the proportions of inferred ancestry estimated from ADMIXTURE [48] and each phecode (see “Methods”; Additional file 5: Table S9). After correcting for the number of tested phecodes, we find numerous significant phecode-ancestry associations: 210 associations within the HL-Other SIRE, 133 within the NH-White SIRE, and 65 within the NH-Asian SIRE, and 16 associations within the NH-AfAm SIRE. Across SIREs, both the top associated phecode categories as well as the direction of the associations greatly vary. Out of the top 3 phecode categories with the most associations in each SIRE group, the most commonly shared group is the endocrine/metabolic category (HL-Other, NH-White, NH-Asian). Even within this category, looking at the statistics quantifying the association of the proportion of European ancestry with endocrine/metabolic phenotypes, there are exclusively 5 negative associations within the NH-White group, 22 negative associations within the HL-Other group (and 2 positive associations), but 5 positive associations and no negative associations within the NH-Asian group. The other top phenotype categories for each SIRE are also unique, where the HL-Other SIRE’s top categories include digestive and respiratory phenotypes, the NH-White SIRE’s top categories include neoplasms and dermatologic phenotypes, and the NH-Asian SIRE’s top categories includes psychiatric and infectious diseases. Specifically, we find that within the HL-Other population, the overall proportion of European ancestry is significantly negatively associated (*p*-value=8.09×10^−10^) with chronic nonalcoholic liver disease and the proportion of Native American ancestry is significantly positively associated (*p*=7.68×10^−9^) (Fig. 6, Additional file 1: Fig. S11), which is consistent with previous studies [71, 72]. These results suggest that not only are some disease statuses associated with SIRE and continental GIA, but the specific ancestry proportions may also correlate with disease risk.

### Genome and phenome-wide association scans identify known risk regions and elucidate correlated phenotypes

EHR-linked biobanks also offer the opportunity of investigating genetic associations with traits across the genome. These efforts impose special challenges, such as adjusting for population stratification and cryptic relatedness in health systems that serve entire families as well as extracting phenotypes from EHR, namely due to inconsistencies in mapping diagnosis codes (ICD codes) to phenotypes and difficulties in defining appropriate controls for specific phenotypes. We perform GWAS on each of the 6 phenotypes within each GIA group. After filtering out analyses with small sample sizes (*N*<50) and analyses where most SNPs failed the regression, we have a total of 17 analyses (see “Methods”; Additional file 6: Table S10). Overall, we find associations are well-calibrated with little evidence of test statistic inflation (median lambda-GC: 1.01). We find a total of 212 genome-wide significant SNPs (*p*-value <5×10^−8^): 77 associations for skin cancer, 1 for ischemic heart disease, and 58 for chronic nonalcoholic liver disease in the EAA GIA group; 1 association for liver/intrahepatic bile duct cancer and 78 for nonalcoholic liver disease in the HL group; and 1 in the EAA group for heart disease (Additional file 7: Table S11). We did not find any genome-wide significant SNPs within the AA GIA group which could be due to the smaller sample size (*N*=1995).

**Fig. 7:**
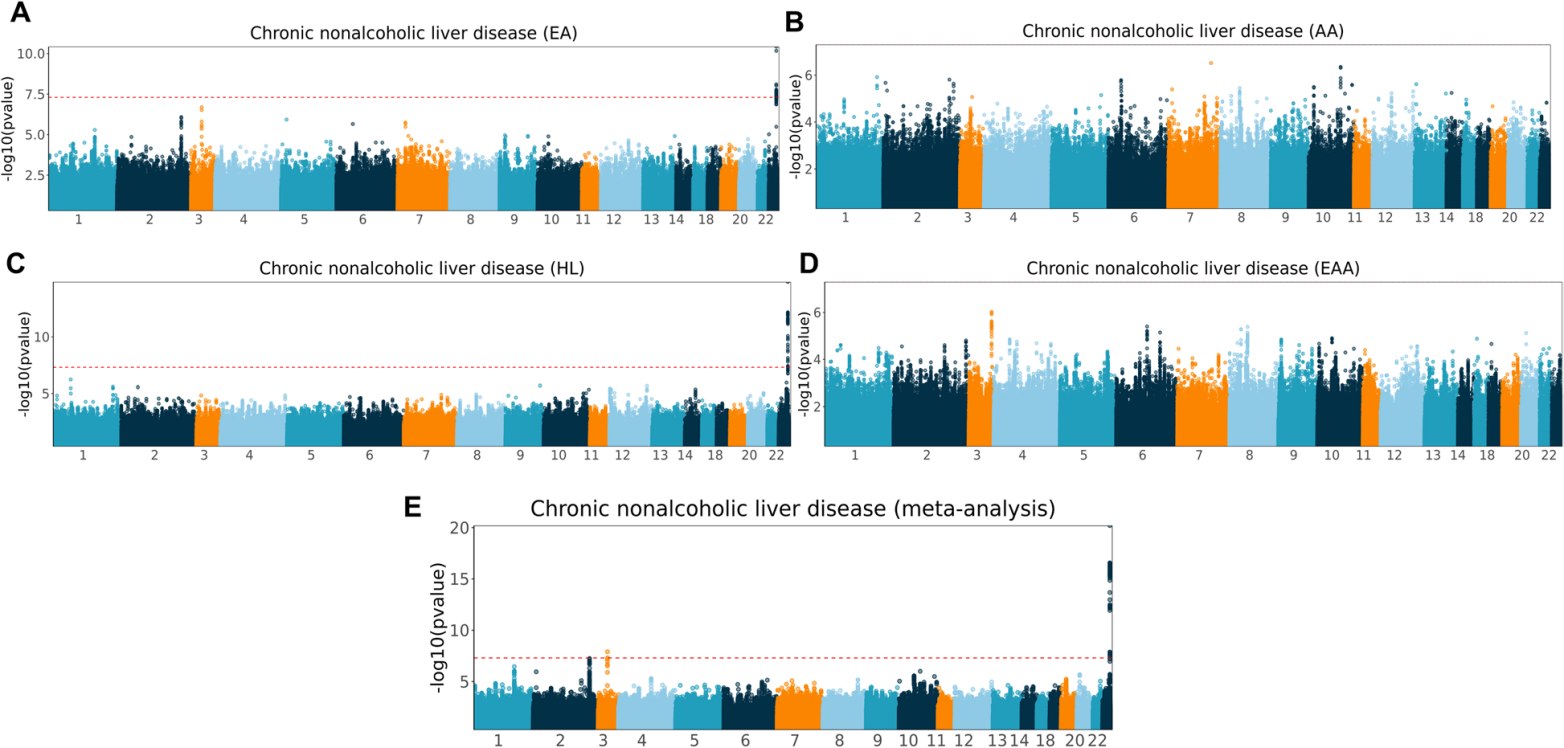
Recapitulating known associations for chronic nonalcoholic liver disease in ancestry-specific and multi-ancestry meta-analyses in ATLAS. GWAS Manhattan plots for chronic nonalcoholic liver disease in the **A** European American, **B** Hispanic Latino American, **C** African American, **D** East Asian American GIA groups in ATLAS, and **E** the meta-analysis performed across all 4 GIA groups. The red dashed line denotes genome-wide significance (*p*-value < *5*×10^-8^). We recapitulate a known association at the 22q13.31 locus

First, we observe ancestry-specific associations, such as a strong association at rs12203592 for skin cancer (*p*-value=2.59×10^−32^) in the European American (EA) GIA group (Additional file 1: Fig. S12). When performing the association for this phenotype in the other GIA groups, we do not have adequate sample size to perform a successful association test at the majority of the SNPs. For the East Asian American (EAA) and the Hispanic Latino (HL) GIA groups, all SNPs resulted in a *p*-value estimate of NA, denoting that the association test had failed. When performing a meta-analysis between the EA and HL GIA groups, we do not find any significant associations despite the strong association originally reported in the EA GIA group. And an analysis within the African American (AA) GIA group was not performed due to low sample size (*N* < 50). We also see significant associations across multiple ancestry groups. For example, in the analyses for nonalcoholic liver disease, we find 58 genome-wide significant SNPs in the EA GIA analysis and 70 in the HL analysis (Fig. 7). All genome-wide significant SNPs from both studies fall within the 22q13.31 locus, which contains the PNPLA3 gene. This gene has been extensively studied for its role in the risk of various liver diseases such as nonalcoholic fatty liver disease [73, 74]. Interestingly, we see more associated SNPs within the Admixed American (*N*-Case=1466) ancestry group despite the larger sample size in the European ancestry group (*N*-Case=3177). The lead SNP from both analyses, rs2294915 (*p*-value(HL)=2.32×10^−16^, *p*-value(EA)=6.73×10^−11^), is an intronic variant in the PNPLA3 gene and has MAF=0.49 in the HL GIA but only MAF=0.24 in the EA GIA which could contribute to the heightened associations in the HL GIA.

We next perform a meta-analysis across all genetic ancestry groups under a fixed effects model for each trait for a total of 6 meta-analyses (Additional file 1: Fig. S12-S17). Meta-analyses present a way to increase statistical power through increased sample size. We observe a total of 11 genome-wide significant associations: 28 for ischemic heart disease (27 new), 82 (14 new) for chronic nonalcoholic liver disease, and 1 (new) association for liver/intrahepatic bile duct cancer. Specifically, 42 of these associations were not found in any of the ancestry-specific analyses, such as the two additional significantly associated regions from the meta-analysis of chronic liver disease (Fig. 7), demonstrating that the added power can identify associations not significant in the ancestry-specific analyses. In the ancestry-specific analyses for heart disease, we only see 1 significant SNP across all ancestry groups that barely reaches genome-wide significance (*p*-value=4.109×10^−8^). After performing the meta-analysis, this increases to a total of 28 significant SNPs all within a locus on chromosome 9, with the top SNP having *p*-value=3.22×10^−10^ (Additional file 1: Fig. S14).

We are not able to perform a meta-analysis for skin cancer since were are limited to only the statistics computed from the EA GIA group (N-Case=4,583, N-Control=17,603, M=6,017,984). The analysis in the AA group was omitted to insufficient sample size (*N*-Case=38, *N*-Control=1923), and all of the SNPs in the HL (*N*-Case=247, *N*-Control=5720) and EAA (*N*-Case=83, *N*-Control=3205) analyses had failed association tests at all SNPs. Specifically, the association tests resulted in a *p*-value estimate of NA which is likely due to the small number of cases or difference in allele frequencies across GIA groups. For example, the minor allele frequencies (MAF) of rs12203592, the top SNP for skin cancer in the EA analysis, greatly vary across GIA groups: MAF-EA=0.17, MAF-AA=0.01, MAF-HL=0.07, MAF-EAA=6.010–4. Thus, populations with a lower MAF of associated variants would require larger sample sizes to have sufficiently powered association tests. This demonstrates that in cases where there are large differences in MAF at associated variations, ancestry-specific analyses would be preferred over a meta-analysis which could actually lead to a reduction in power.

**Fig. 8:**
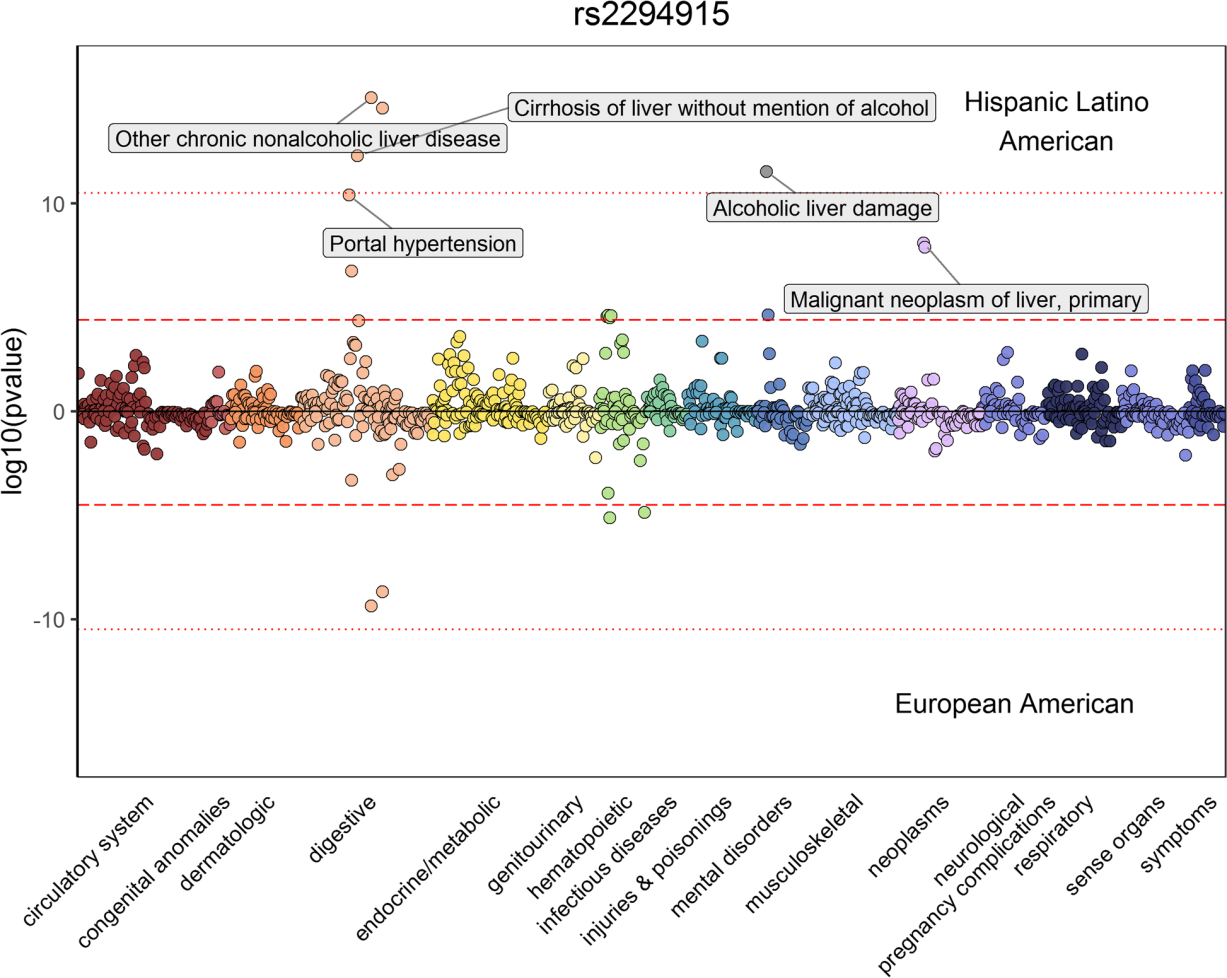
Identifying correlated phenotypes at rs2294915 in both the Hispanic Latino American and European American GIA groups in ATLAS. We show a PheWAS plot at rs2294915 for the Hispanic Latino American (top) and European American (bottom) GIA groups. The red dashed line denotes *p*-value=4.09×10^−5^, the significance threshold after adjusting for the number of tested phenotypes. The red dotted line denotes the significance threshold after correcting for both genome-wide significance and the number of tested phenotypes (*p*-value=4.09×10^−11^)

Next, we investigated the top significant association for each phenotype and GIA group through a PheWAS. For rs12203592, an intron variant in the IRF4 gene, we observe significantly associated phenotypes related to skin cancer such as actinic keratosis and basal cell carcinoma in the EA GIA analysis, both of which have been identified in previous PheWAS studies [33] (Additional file 1: Fig. S18A). At rs1333045, the top SNP associated with ischemic heart disease in the EA GIA analysis, we find related phenotypes such as coronary atherosclerosis and angina pectoris (Additional file 1: Fig. S18B). We also perform a PheWAS at rs2294915, the lead SNP for liver/intrahepatic bile duct cancer in the HL GIA analysis and the lead SNP in the analyses for chronic nonalcoholic liver disease in both the EA and HL GIA analyses (Fig. 8). We find that multiple neoplastic and neurologic phenotypes reach significance exclusively in the HL analysis. These groups of phenotypes are consistent with the known comorbidities with severe liver disease [75–77]. Performing a power analysis on the effective sample sizes [51] of the associated phenotypes in both GIA groups, we do not find evidence that the observed effects are solely due to sample size (see “Methods”; Additional file 8: Table S12). Overall, these findings suggest possible differential genetic architecture across these two populations, as well as variation even at the phenotype level, reflecting possible genetic or environmental modifiers of important comorbidities.

## Discussion

As the field moves forward with increased collaboration between the genetics and healthcare communities, it is of utmost importance to also be aware of potential pitfalls that may occur when translating research findings into actual clinical populations. Currently, many clinical protocols implicitly perpetuate racial bias [23, 78–81]. Many of these flawed policies stemmed from erroneously linking race, a social rather than biological construct, with disease risk despite not presenting any biological justification. Although race and genetic ancestry are correlated [82, 83], our work shows that populations constructed from these two concepts are not analogous. We encourage protocol decisions that are rooted in concrete biological phenomena whenever possible, such as genetic markers, providing transparent, immutable criteria. For example, Benign Ethnic Neutropenia (BEN) is observed predominantly in African Americans, but specifically is strongly associated with the variant at rs2814778 [84, 85]. Recent studies have suggested that genotype screening at rs2814778 could aid in the interpretation of neutropenia in African Americans and avoid unnecessary invasive procedures as well as lead to an increase of the inclusion of these individuals to various treatments [86].

However, in practice, genetic information is not easily accessible for patients at all institutions. Additionally, certain disease prediction-based algorithms that leverage SIRE may be favorable to the non-adjusted version. SIRE is correlated with genetic ancestry as well as other disease risk factors (sociocultural, socioeconomic, and geographic), making it straightforward and more easily accessible to add valuable information into models without explicit measurements. We recommend deliberately considering the potential harm versus benefit of using SIRE-adjusted prediction models in each use-case. The practice of race/ethnicity-guided algorithms and guidelines inherently reinforces the idea of race-based medicine and embeds the idea that health inequities also stem from biological differences. It is an ongoing discussion about whether or not the inclusion of race/ethnicity information actually re-allocates resources away from racial/ethnic minority patients, causing more harm and an increase in health inequities.

There are various limitations within our study, and we describe a few of these in detail as follows. First, the phecodes are based on ICD codes, and due to the nature of billing codes, this form of labeling does not constitute a formal patient diagnosis and may contain individuals who do not have the specific disease. We also only require the presence of one phecode to define a phenotype which is a significant assumption. Although we present exploratory analyses assessing the role of phecode occurrence when defining phenotypes, we underscore that this imprecise phenotyping limits the power of our study. For further investigation into specific phenotypes, we recommend refining each phenotype definition based on additional disease-specific factors and metrics. For example, one could incorporate additional EHR features, such as those described by the algorithms in the PheKB database [70]. Although ICD codes are an international standard, the accuracy of phecode assignment could differ considerably due to heterogeneity in billing practices across medical centers, hospitals, and clinics both within the UCLA Health System as well as across other institutions. This heterogeneity could present future challenges when replicating studies or porting algorithms to other institutions. Second, due to the de-identified nature of the data, we lack information that could help us better describe the fine-scale population groups. For example, birthplace, zip code, and family history have been shown to be useful descriptors for determining subgroups of genetic ancestry [56]. Geographic information could also be used as a proxy for various environmental exposures such as pollution. Additional socioeconomic information, such as income and availability of health insurance, could likely account for a portion of observed associations as well as provide more insight into the socioeconomic determinants of health. Third, our findings within the African and South Asian ancestry populations are limited due to the smaller sample sizes. As sample sizes increase, we hope to further refine population substructure within these initial continental ancestry groups and have the power to detect novel disease associations that have previously been mired by lack of statistical power.

An open question and potential additional limitation of this work is generalizing these results to broader populations that extend beyond the UCLA Health system. For example, even when assessing self-identified race/ethnicity statistics, there is a discrepancy between the breakdown of SIRE within ATLAS and the city of Los Angeles. For example, it is reported that 48.5% of residents of Los Angeles self-identify as Hispanic or Latino [16], compared to only 15.96% of individuals in ATLAS. This could be due to the specific location of the UCLA Health system which consists of hospitals both in West Los Angeles and Santa Monica which are both located in affluent neighborhoods. When comparing demographic data recorded for the city of Santa Monica, which could be a more accurate representation of the area surrounding the UCLA hospitals as opposed to Los Angeles as whole, we find that 15.4% of individuals self-identify as Hispanic or Latino [87]. Overall, the distribution of the majority of racial groups in Santa Monica have a high concordance with those reported in ATLAS (Additional file 2: Tables S1, S2, S3). Since travel to treatment centers is often a barrier to treatment [88], this might explain why the ATLAS population mostly captures the demographics of the nearby areas. Furthermore, previous work has shown that referral rates for some types of procedures vary disproportionately across race and ethnic groups [89–91]. As a tertiary and quaternary referral center, this pattern could be reflected in the UCLA patient population. In addition, this discrepancy could specifically reflect the variations in patient participation rate across demographic groups. Previous studies have shown that trust in the health system and medical community is a large factor when patients consider whether to participate in medical research [92–94]. Overall, there are a myriad of factors that influence the population and health of a specific region such as socioeconomic status, political geography, immigration, and historical events—the majority of these not being race neutral. These observations suggest that many of these analyses should be interpreted with respect to the UCLA Health system specifically and extrapolating results to larger geographic areas or groups as a whole, should be done with caution.

## Conclusions

In this work, we introduce the ATLAS Community Health Initiative, a biobank embedded within the UCLA Health medical system consisting of de-identified EHR-linked genomic data from a diverse patient population. The UCLA Health system serves Los Angeles County, leading to a study population of great demographic, genetic, and phenotypic diversity. We investigate ancestry both on the continental as well as the subcontinental population level and find that genetic ancestry and self-reported demographic information yield distinct subpopulations in the ATLAS biobank. We present a collection of results cataloging the associations between genetically inferred ancestry and EHR-derived phenotypes where we find that disease status is not only associated with continental genetic ancestry but also associated with the specific admixture profile describing an individual. We use multi-ancestry pipelines to recapitulate known associations for chronic nonalcoholic liver disease at the 22q13.31 locus and perform a PheWAS at the lead SNP, where we find associations with neurologic and neoplastic phenotypes exclusively in the HL GIA group. As the sample size increases, the ATLAS Community Health Initiative will enable rigorous genetic and epidemiological studies to further understand the role of genetic ancestry in disease etiology, with a specific aim to accelerate genomic medicine in diverse populations. Already, the ATLAS biobank accounted for 73.4% of the Admixed American samples utilized in the primary analysis from the COVID-19 Host Genetics Initiative [95].

We conclude by discussing directions for future work. Although we investigate admixed populations, such as African American and Hispanic/Latino populations, admixed individuals who do not fall under these groups are excluded from downstream analyses due to concerns over population structure. In the future, we hope to incorporate methods and pipelines that properly control for population structure in all types of admixed populations. Additionally, we plan to compute polygenic risk scores (PRS) across all 5 continental ancestry groups. PRS has already shown modest clinical utility for diseases such as breast cancer [96] and cardiovascular disease [97], but has proven difficult to perform accurate predictions across populations [13]. The genetic diversity within the ATLAS Community Health Initiative biobank partnered with the longitudinal clinical data provides a unique resource to further explore the role of ancestry in PRS prediction. Furthermore, as the size of the biobank grows and more data is collected over time, we hope to explore even more individualized health solutions and interventions.

## Supplementary Information

**Additional file 1.** Supplementary Materials providing additional graphs and figures.

**Additional file 2: Table S1.** Self-identified race. **Table S2.** Self-identified ethnicity. **Table S3.** Self-identified race/ethnicity (SIRE). **Table S4.** Genetically inferred ancestry. **Table S5.** Concordance between SIRE and genetically inferred ancestry, **Table S6.** Average ADMIXTURE proportions stratified by SIRE.

**Additional file 3: Table S7.** Associations between genetically inferred ancestry and phecodes within ATLAS.

**Additional file 4: Table S8.** Associations between genetically inferred ancestry and phecodes within ATLAS while adjusting for SIRE.

**Additional file 5: Table S9.** Associations between genetic ancestry proportions and phecodes within SIREs.

**Additional file 6: Table S10.** Summary of GWAS analyses in ATLAS.

**Additional file 7: Table S11.** Genome-wide significant associations in ATLAS.

**Additional file 8: Table S12.** Effective sample sizes and effect sizes of correlated phenotypes for chronic nonalcoholic liver disease. **Table S13.** Role of phecode occurrences for defining cases. **Table S14.** Summary of methods. **Table S15.** Comparison of GIA clusters when restricting to unrelated individuals. **Table S16**. Comparison of genetic ancestry proportions within SIREs when using unrelated individuals.

## Data Availability

All PheWAS summary statistics are publicly available and are available in the ATLAS PheWAS repository, https://atlas-phewas.mednet.ucla.edu/. GWAS summary statistics from this study can be accessed in the GWAS Catalog under the following accession IDs: GCST90128454, GCST90128455, GCST90128456, GCST90128457, GCST90128458, GCST90128459, GCST90128460, GCST90128461, GCST90128462, GCST90128463, GCST90128464, GCST90128465, GCST90128466, GCST90128467 [98]. See https://www.ebi.ac.uk/gwas/studies/[accession_ID]. (e.g. https://www.ebi.ac.uk/gwas/studies/GCST90128455).

## Abbreviations

GWAS: Genome-wide association studies
PheWAS: Phenome-wide association studies
SNP: Single-nucleotide polymorphism
GIA: Genetically inferred ancestry
SIRE: Self-identified race/ethnicity
EA: European American
AA: African American
HL: Hispanic Latino
EAA: East Asian American
SAA: South Asian American
