# Author Correction: Leveraging genomic diversity for discovery in an electronic health record linked biobank: the UCLA ATLAS Community Health Initiative

**DOI:** 10.1186/s13073-022-01128-5

**Published:** 2022-11-16

**Authors:** Ruth Johnson, Yi Ding, Vidhya Venkateswaran, Arjun Bhattacharya, Kristin Boulier, Alec Chiu, Sergey Knyazev, Tommer Schwarz, Malika Freund, Lingyu Zhan, Kathryn S. Burch, Christa Caggiano, Brian Hill, Nadav Rakocz, Brunilda Balliu, Christopher T. Denny, Jae Hoon Sul, Noah Zaitlen, Valerie A. Arboleda, Eran Halperin, Sriram Sankararaman, Manish J. Butte, UCLA Precision Health Data Discovery Repository Working Group, UCLA Precision Health ATLAS Working Group, Clara Lajonchere, Daniel H. Geschwind, Bogdan Pasaniuc

**Affiliations:** 1 Department of Computer Science, University of California, Los Angeles, Los Angeles, CA 90095 USA; 2 Department of Pathology and Laboratory Medicine, David Geffen School of Medicine, University of California, Los Angeles, Los Angeles, CA 90095 USA; 3 Bioinformatics Interdepartmental Program, University of California, Los Angeles, Los Angeles, CA 90095 USA; 4 Department of Oral Biology, School of Dentistry, University of California, Los Angeles, Los Angeles, CA 90095 USA; 5 Institute for Quantitative and Computational Biosciences, David Geffen School of Medicine, University of California, Los Angeles, Los Angeles, CA 90095 USA; 6 Department of Medicine, Division of Cardiology, University of California, Los Angeles, Los Angeles, CA 90095 USA; 7 Department of Human Genetics, David Geffen School of Medicine, University of California, Los Angeles, Los Angeles, CA 90095 USA; 8 Department of Genetics, Stanford School of Medicine, Stanford, CA 94305 USA; 9 Molecular Biology Institute, David Geffen School of Medicine, University of California, Los Angeles, Los Angeles, CA 90095 USA; 10 Program in Neurogenetics, Department of Neurology, David Geffen School of Medicine, University of California, Los Angeles, Los Angeles, CA 90095 USA; 11 Department of Computational Medicine, David Geffen School of Medicine, University of California, Los Angeles, Los Angeles, CA 90095 USA; 12 Division of Hematology/Oncology, Department of Pediatrics, Gwynne Hazen Cherry Memorial Laboratories, University of California, Los Angeles, Los Angeles, CA 90095 USA; 13 Molecular Biology Institute, University of California, Los Angeles, Los Angeles, CA 90095 USA; 14 Jonsson Comprehensive Cancer Center, University of California, Los Angeles, Los Angeles, CA 90095 USA; 15 Department of Psychiatry and Biobehavioral Sciences, University of California, Los Angeles, Los Angeles, CA 90095 USA; 16 Department of Anesthesiology and Perioperative Medicine, David Geffen School of Medicine, University of California, Los Angeles, Los Angeles, CA 90095 USA; 17 Department of Pediatrics, David Geffen School of Medicine, University of California, Los Angeles, Los Angeles, CA 90095 USA; 18 Institute of Precision Health, University of California, Los Angeles, Los Angeles, CA 90095 USA

**Correction: Genome Med 14, 104 (2022)**

**https://doi.org/10.1186/s13073-022-01106-x**

The original publication of this article [1] contained incorrect figure panels/labels in Figs. 2 and 5 and Additional file 1.

**Fig. 2:**
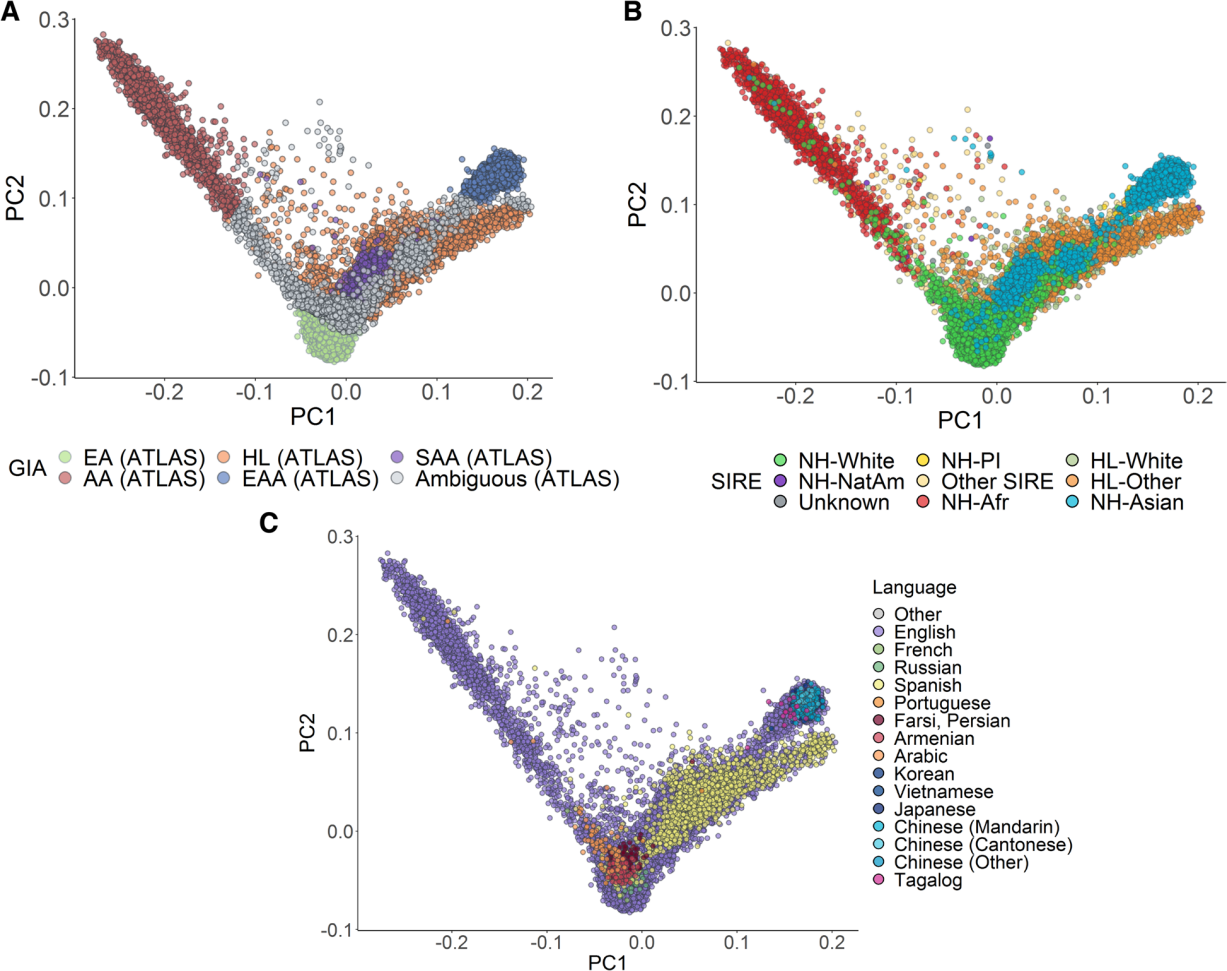
Global PCA reflects self-identified race/ethnicity and language of ATLAS participants. **A** Genetic PCs 1 and 2 of individuals in ATLAS (*N*=36,736) shaded by continental GIA as inferred from 1000 Genomes. **B**, **C** The first two genetic PCs of the ATLAS participants shaded by SIRE and preferred language, respectively. To improve visualization in **C**, only languages with >10 responses were assigned a color

**Fig. 5:**
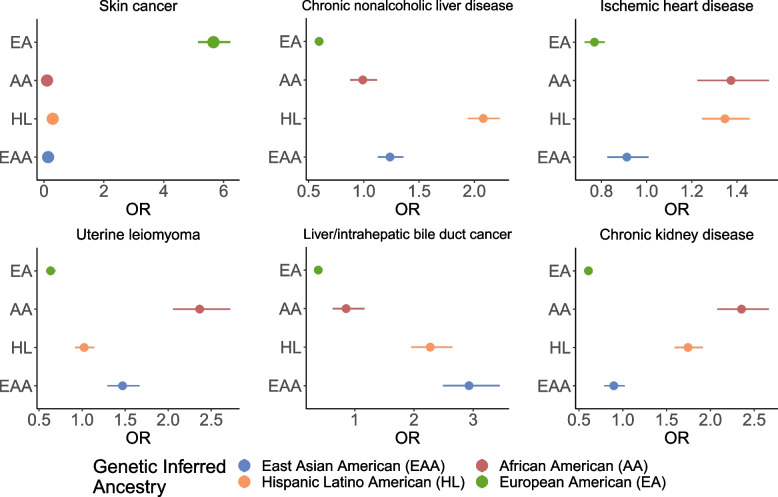
Disease associations vary across continental genetically inferred ancestry groups in ATLAS. We show the odds ratio computed from associating each phenotype with individuals’ genetically inferred ancestry in ATLAS (*N*=36,736) under a logistic regression model. Error bars represent 95% confidence intervals

The correct figures are available in this correction article. The original article has been updated to correct these errors.

## Supplementary Information

**Additional file 1: Figure S1.** Self-identified race/ethnicity (SIRE) and genetically inferred ancestry (GIA) capture distinct information. We show the percentage breakdown of (A) SIREs and (B) continental genetic ancestry for all individuals in ATLAS (*N*=36,736). We exclude individuals whose self-identify race and/or ethnicity are unknown. **Figure S2.** Clustering individuals by continental GIA using PCA and K-nearest neighbors clustering. Genetic PCs 1-6 of ATLAS participants (*N*=36,736) and individuals in 1000 Genomes stratified by genetic ancestry groups. **Figure S3.** PCA in the East Asian American GIA group by self-identified race and language. (A) Genetic PCs 3 and 4 from principal component analysis performed on East Asian American GIA group (*N*=3,331) colored by self-identified race and (B) self-identified preferred language. Only languages with >10 responses are assigned a color. **Figure S4.** East Asian American GIA subclusters. Principal component analysis on the East Asian American GIA group in ATLAS with East Asian ancestry samples from 1000 Genomes. (A) shows the projection of PCs 3 and 4 and subclusters identified from performing K-nearest neighbors using population labels from 1000 Genomes to define clusters and (B) self-identified race information from ATLAS as cluster labels. **Figure S5.** PCA in the European American GIA group by self-identified race and language. (A) Genetic PCs 3 and 5 from principal component analysis performed within the European American GIA group (*N*=22,380) colored by self-identified race and (B) self-identified preferred language. Only languages with >10 responses are assigned a color. **Figure S6.** PCA in the Hispanic Latino American GIA group by self-identified ethnicity, language, and inferred ancestry proportions. (A) Genetic PCs 1 and 2 from principal component analysis performed within the Hispanic Latino American GIA group (*N*=6,073) colored by self-identified ethnicity and (B) self-identified preferred language. Only languages with >10 responses are assigned a color. (C) and (D) show the PCs shaded according to the estimated proportion of European and Native American genetic ancestry inferred from ADMIXTURE. **Figure S7.** PCA in the African American GIA group by genetic ancestry, self-identified race, language, and inferred ancestry proportions. Genetic PCs 1 and 2 from principal component analysis performed within the Hispanic Latino American GIA group (*N*=1995) colored by (A) genetic ancestry of individuals from 1000 Genomes, (B) self-identified race and (B) self-identified preferred language. Only languages with >10 responses are assigned a color. (C) and (D) show the PCs shaded according to the estimated proportion of European and African genetic ancestry inferred from ADMIXTURE. **Figure S8.** Individual admixture proportions vary across and within SIRE. Admixture proportions for ATLAS participants (*N*=36,736) were estimated using ADMIXTURE with k=4, 5, or 6 ancestral populations. Within each SIRE, we visualize the proportions of each ancestry as a vertical bar for each individual. Individuals are ordered on the x-axis by global ancestry proportions. For k=4, the respective components correspond to European, African, East Asian, and Native American ancestries. **Figure S9.** Disease associations vary across continental GIA groups in ATLAS even after adjusting for SIRE. We show the odds ratio computed from associating each phenotype with individuals’ genetically inferred ancestry in ATLAS (*N*=36,736) under a logistic regression model after accounting for each individual’s SIRE category. Error bars represent 95% confidence intervals. **Figure S10.** Disease associations vary across subcontinental groups within the East Asian American GIA group. For individuals in the East Asian American GIA group in ATLAS (*N*=3,331), we show the odds ratio computed from associating each phenotype with individuals’ subcontinental GIA group under a logistic regression model. We limit analyses to phenotypes with N>20 cases; for this reason, the analysis for skin cancer has been omitted. Error bars represent 95% confidence intervals. **Figure S11.** Global ancestry correlates with disease prevalence in admixed individuals. Individuals by SIRE who have had a diagnosis of (A) skin cancer, (B) chronic kidney disease, or (C) heart disease are binned by their proportions of either European, African, or Native American ancestry estimated using ADMIXTURE. Within each bin, we plot the prevalence of the diagnoses and provide standard errors (+/-1.96 SE) of the computed frequencies. **Figure S12.** Manhattan plot for ancestry-specific analysis for skin cancer. GWAS Manhattan plot for skin cancer in the European American GIA group. The red dashed line denotes genome-wide significance (*p*-value<5× 10^-8^). **Figure S13.** Manhattan plots for ancestry-specific and multi-ancestry meta-analysis for chronic nonalcoholic liver disease. GWAS Manhattan plots for chronic nonalcoholic liver disease in the (A) European American, (B) African American, (C) Hispanic Latino American, (D) East Asian American GIA groups, and (E) the meta-analysis across all GIA groups. The red dashed line denotes genome-wide significance (*p*-value<5× 10^-8^). **Figure S14.** Manhattan plots for ancestry-specific and multi-ancestry meta-analysis for ischemic heart disease. GWAS Manhattan plots for ischemic heart disease in the (A) European American, (B) African American, (C) Hispanic Latino American, and (D) East Asian American GIA groups, and (E) the meta-analysis across all GIA groups. The red dashed line denotes genome-wide significance (*p*-value<5× 10^-8^). **Figure S15.** Manhattan plot for ancestry-specific analysis for uterine leiomyoma. GWAS Manhattan plots for uterine leiomyoma in the African American GIA group. The red dashed line denotes genome-wide significance (*p*-value<5× 10^-8^). **Figure S16.** Manhattan plots for ancestry-specific and multi-ancestry meta-analysis for liver/intrahepatic bile duct cancer. GWAS Manhattan plots for liver/intrahepatic bile duct cancer in the (A) Hispanic Latino American, (B) East Asian American GIA groups, and (C) the meta-analysis across both GIA groups. The red dashed line denotes genome-wide significance (*p*-value<5× 10^-8^). **Figure S17.** Manhattan plots for ancestry-specific and multi-ancestry meta-analysis for chronic kidney disease. GWAS Manhattan plots for chronic kidney disease in the (A) European American, (B) African American, and (C) the meta-analysis across GIA groups. The red dashed line denotes genome-wide significance (*p*-value<5× 10^-8^). **Figure S18.** PheWAS at top GWAS associations. We show a PheWAS plot at rs12203592 (chr6:396321) and rs1333045 (chr9:22119196) computed within the European American GIA group. The red dashed line denotes *p*-value=4.09× 10^-5^, the significance threshold after adjusting for the number of tested phenotypes. The red dotted line denotes the significance threshold after correcting for both genome-wide significance and the number of tested phenotypes (*p*-value=4.09× 10^-11^). **Figure S19.** Role of phecode occurrences for defining cases. We show the percentage of cases retained while varying the number of required phecode occurrences (x-axis) for 6 phenotypes. In A), phecodes are derived from all types of encounters. In B), phecodes are only derived only from appointments and office, hospital, or procedure visits. The y-axis ranges from 0.95 to 1.0.
