# Supplementary material for: Leveraging genomic diversity for discovery in an electronic health record linked biobank: the UCLA ATLAS Community Health Initiative
Source: Genome Med. 2022 Sep 9;14:104. doi: 10.1186/s13073-022-01106-x (PMC9461263; doi:10.1186/s13073-022-01106-x)
Supplement: Supplementary file 1 — Additional file 1. Supplementary Materials providing additional graphs and figures. [file 13073_2022_1106_MOESM1_ESM.pdf]

Supplementary Materials

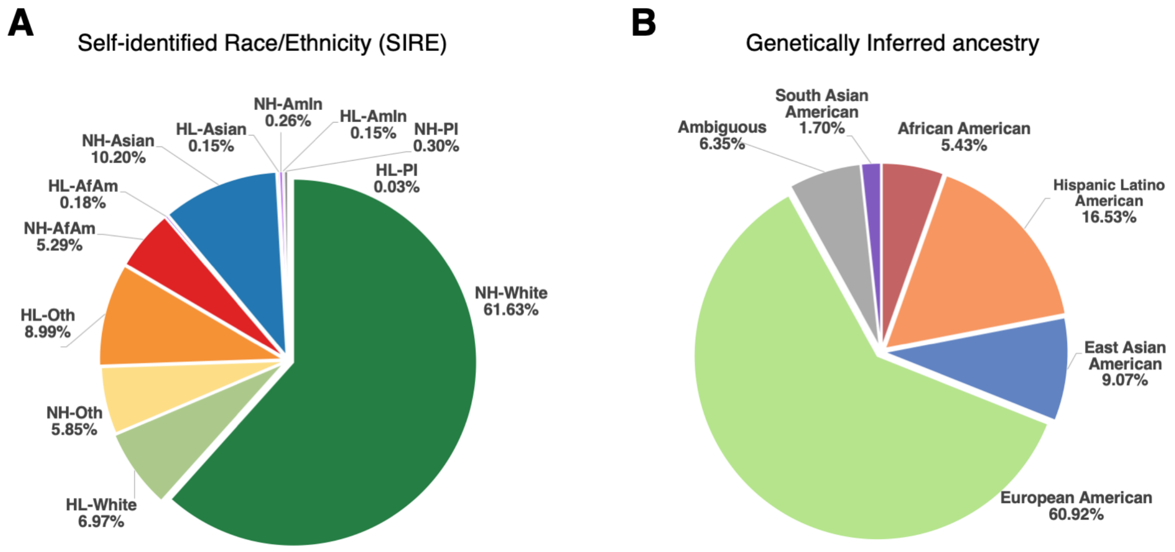

Fig. S1: Self-identified race/ethnicity (SIRE) and genetically inferred ancestry (GIA) capture distinct information. We show the percentage breakdown of (A) SIREs and (B) continental genetic ancestry for all individuals in ATLAS (N=36,736). We exclude individuals whose self-identify race and/or ethnicity are unknown.

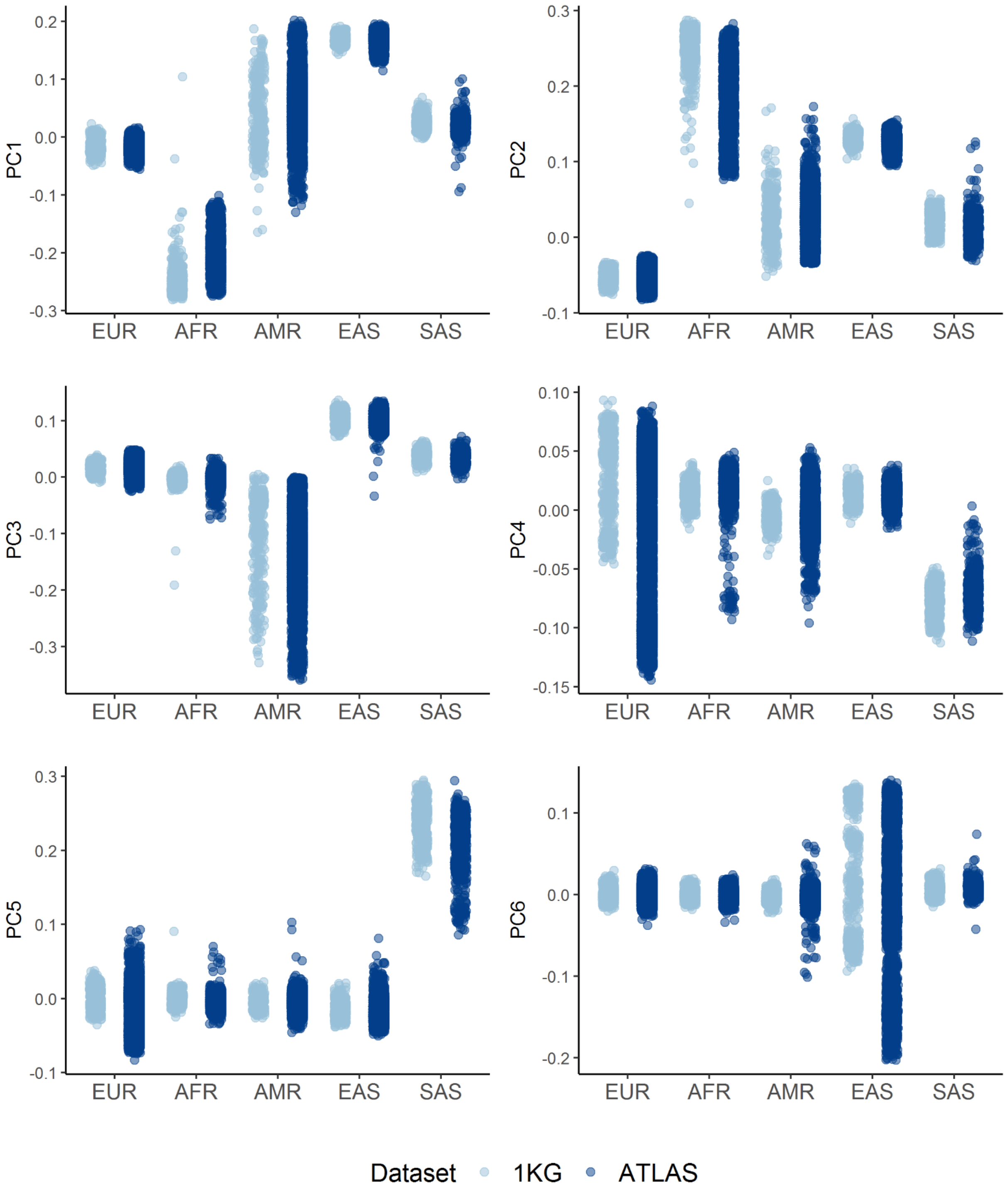

Fig. S2: Clustering individuals by continental GIA using PCA and K-nearest neighbors clustering. Genetic PCs 1-6 of ATLAS participants (N=36,736) and individuals in 1000 Genomes stratified by genetic ancestry groups.

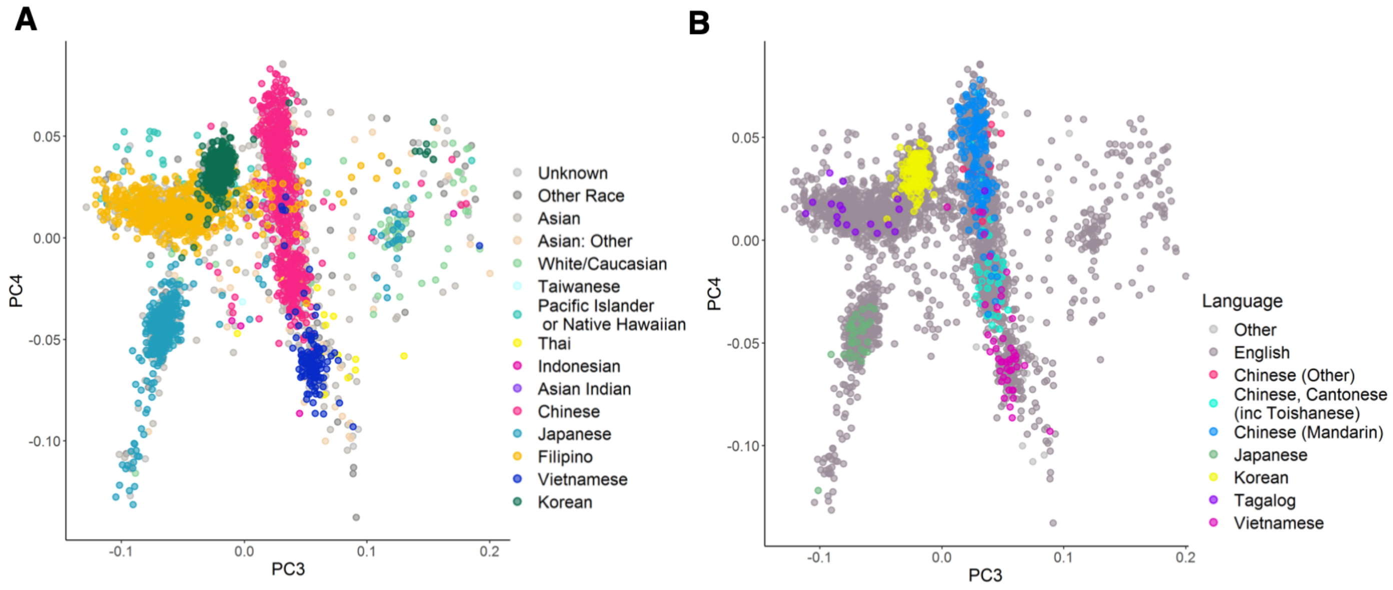

Fig. S3: PCA in the East Asian American GIA group by self-identified race and language. (A) Genetic PCs 3 and 4 from principal component analysis performed on East Asian American GIA group (N=3,331) colored by self-identified race and (B) self-identified preferred language. Only languages with >10 responses are assigned a color.

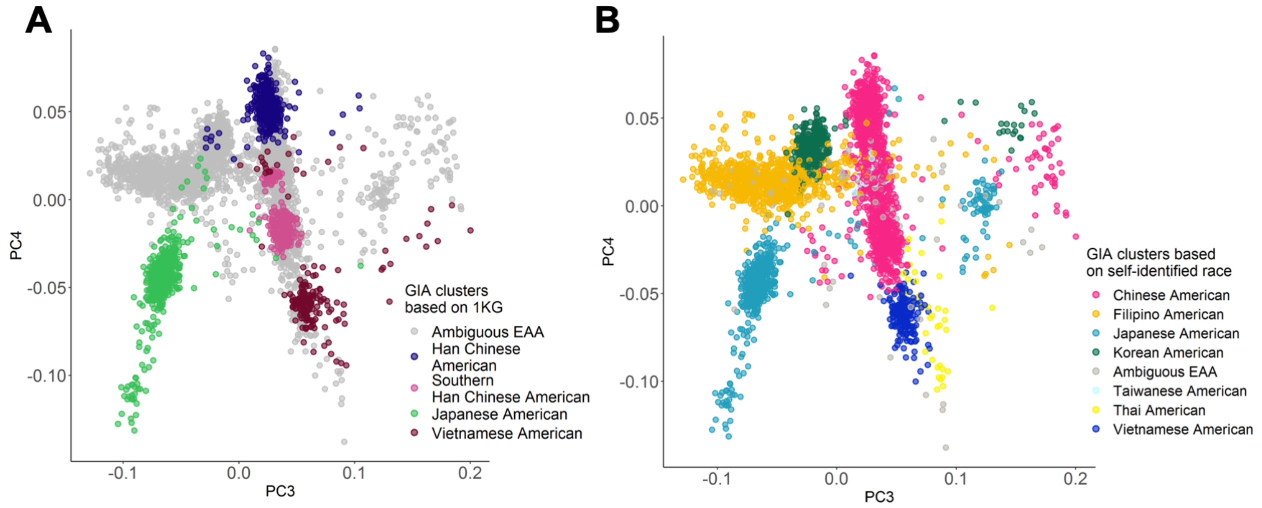

Fig. S4: East Asian American GIA subclusters. Principal component analysis on the East Asian American GIA group in ATLAS with East Asian ancestry samples from 1000 Genomes. (A) shows the projection of PCs 3 and 4 and subclusters identified from performing K-nearest neighbors using population labels from 1000 Genomes to define clusters and (B) self-identified race information from ATLAS as cluster labels.

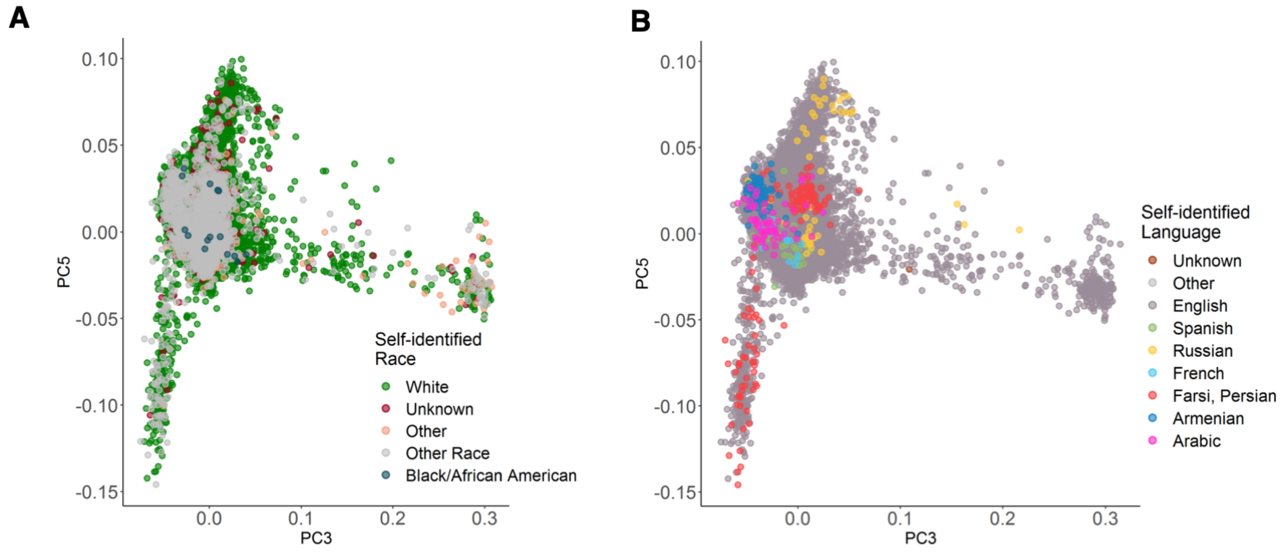

Fig. S5: PCA in the European American GIA group by self-identified race and language. (A) Genetic PCs 3 and 5 from principal component analysis performed within the European American GIA group (N=22,380) colored by self-identified race and (B) self-identified preferred language. Only languages with >10 responses are assigned a color.

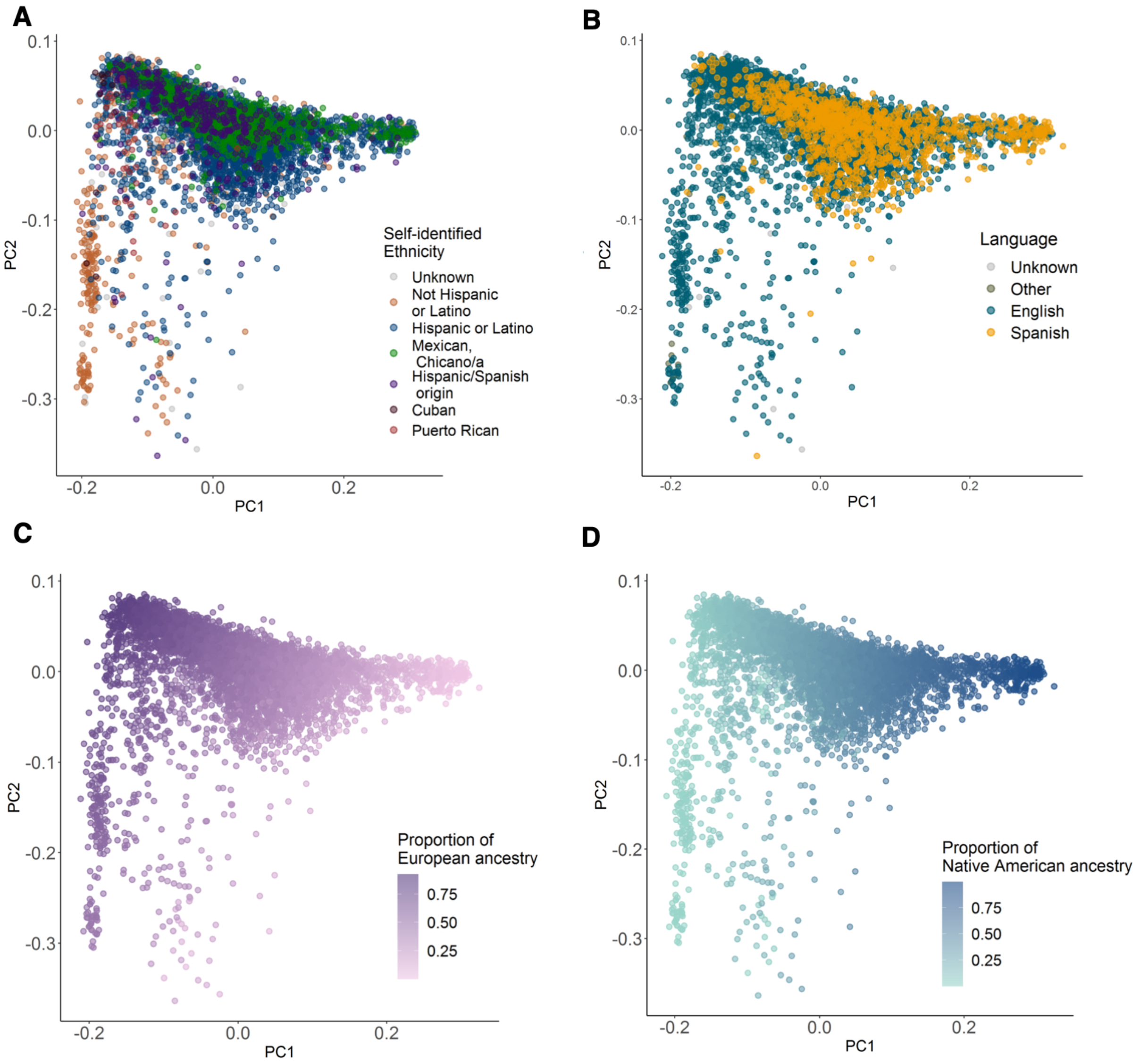

Fig. S6: PCA in the Hispanic Latino American GIA group by self-identified ethnicity, language, and inferred ancestry proportions. (A) Genetic PCs 1 and 2 from principal component analysis performed within the Hispanic Latino American GIA group (N=6,073) colored by self-identified ethnicity and (B) self-identified preferred language. Only languages with >10 responses are assigned a color. (C) and (D) show the PCs shaded according to the estimated proportion of European and Native American genetic ancestry inferred from ADMIXTURE.

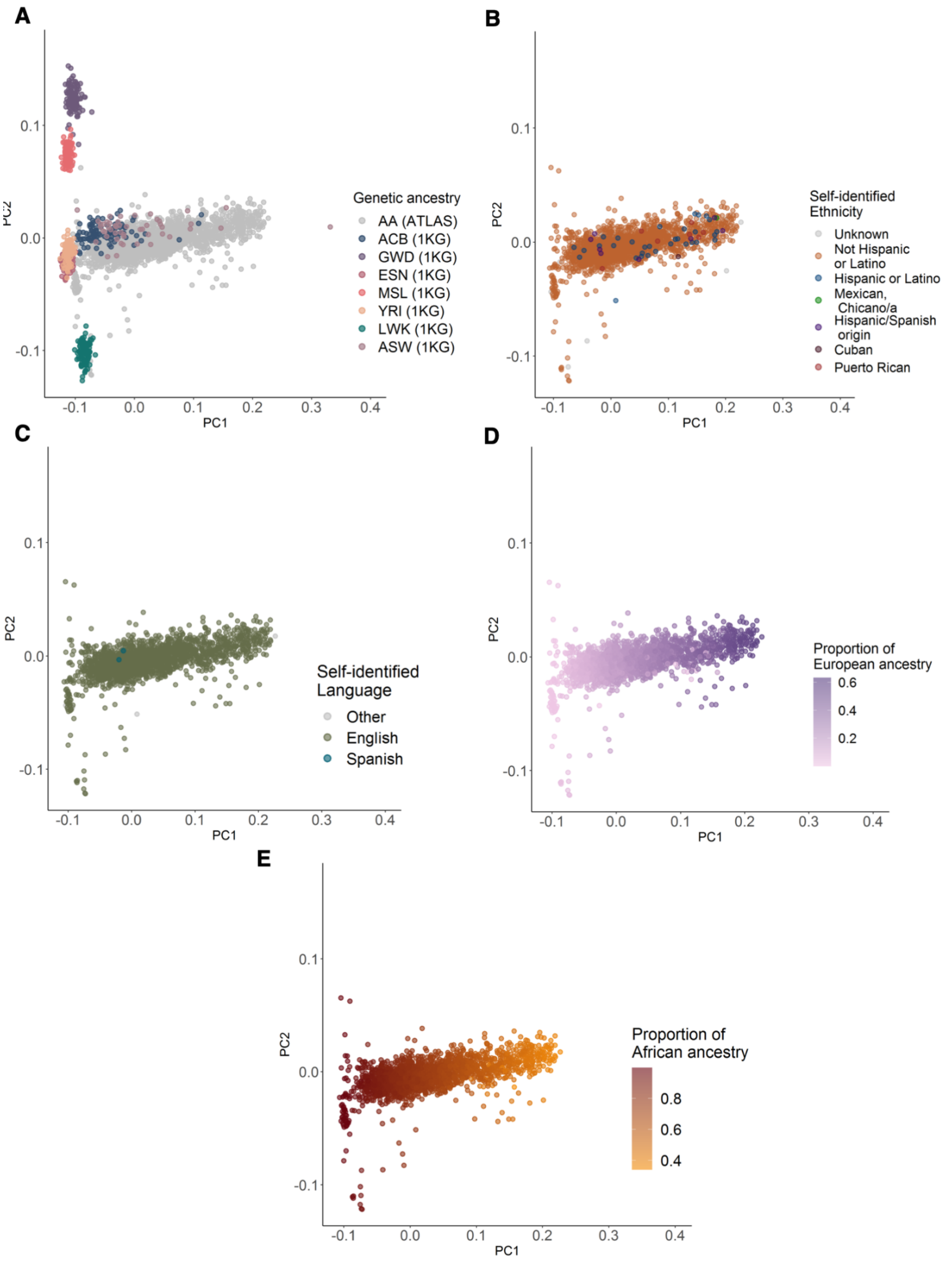

Fig. S7: PCA in the African American GIA group by genetic ancestry, self-identified race, language, and inferred ancestry proportions. Genetic PCs 1 and 2 from principal component analysis performed within the Hispanic Latino American GIA group (N=1995) colored by (A) genetic ancestry of individuals from 1000 Genomes, (B) self-identified race and (B) self-identified preferred language. Only languages with >10 responses are assigned a color. (C) and (D) show the PCs shaded according to the estimated proportion of European and African genetic ancestry inferred from ADMIXTURE.

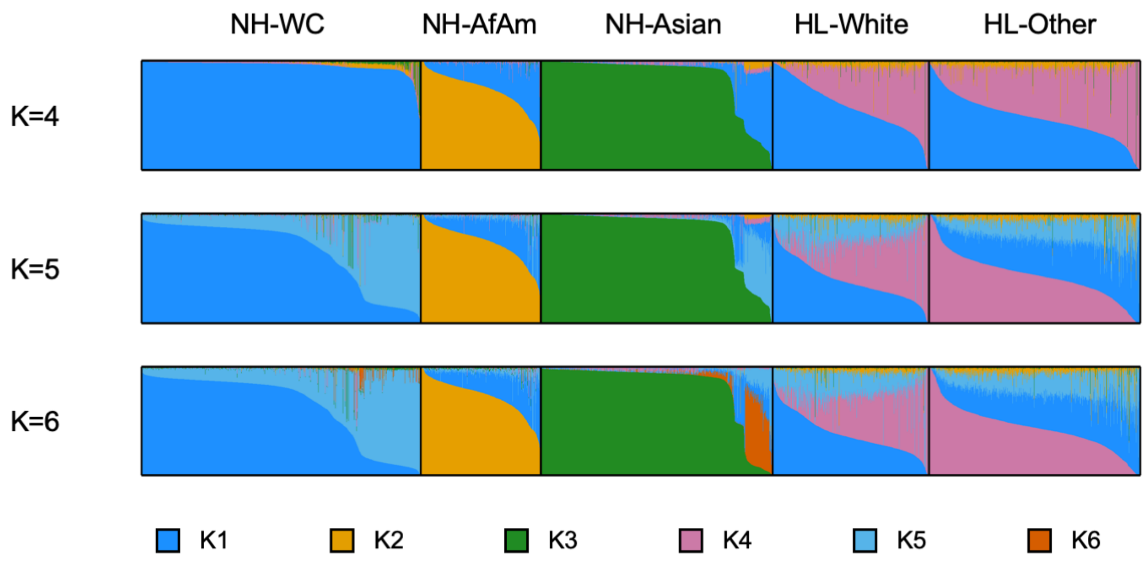

Fig. S8: Individual admixture proportions vary across and within SIRE. Admixture proportions for ATLAS participants (N=36,736) were estimated using ADMIXTURE with k=4, 5, or 6 ancestral populations. Within each SIRE, we visualize the proportions of each ancestry as a vertical bar for each individual. Individuals are ordered on the x-axis by global ancestry proportions. For k=4, the respective components correspond to European, African, East Asian, and Native American ancestries.

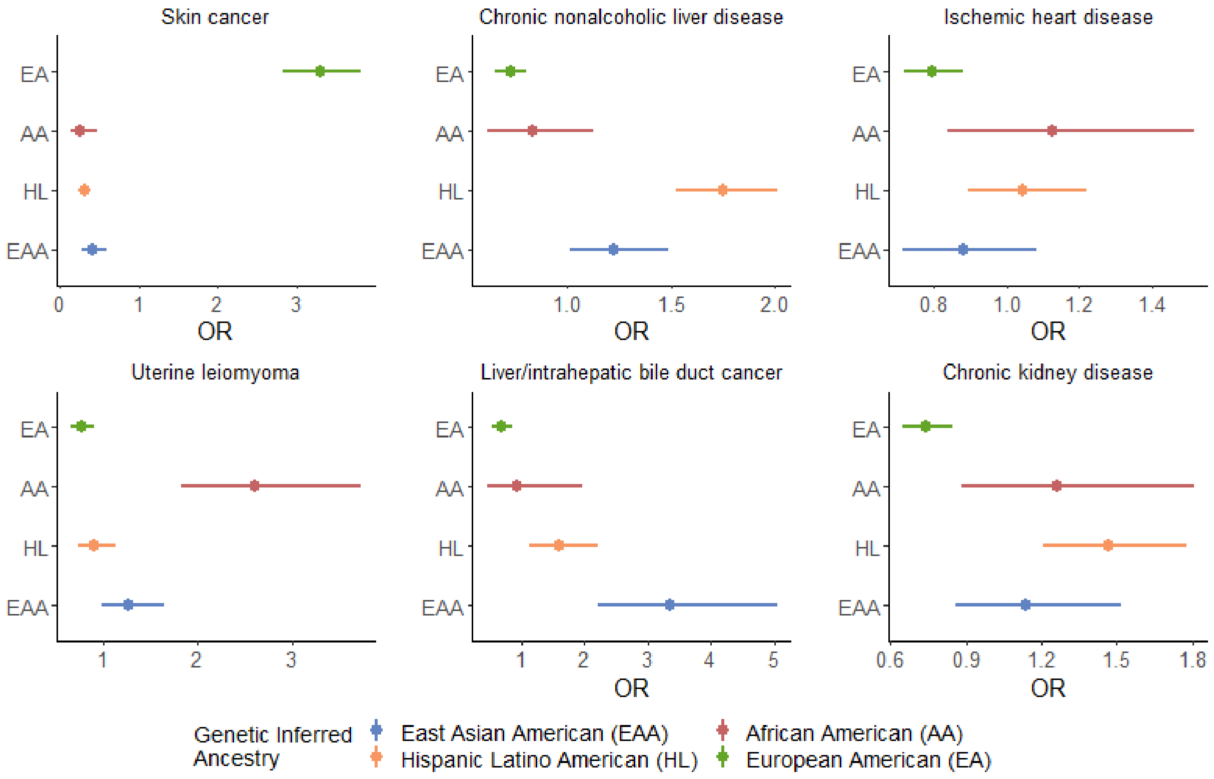

Fig. S9: Disease associations vary across continental GIA groups in ATLAS even after adjusting for SIRE. We show the odds ratio computed from associating each phenotype with individuals' genetically inferred ancestry in ATLAS (N=36,736) under a logistic regression model after accounting for each individual's SIRE category. Error bars represent 95% confidence intervals.

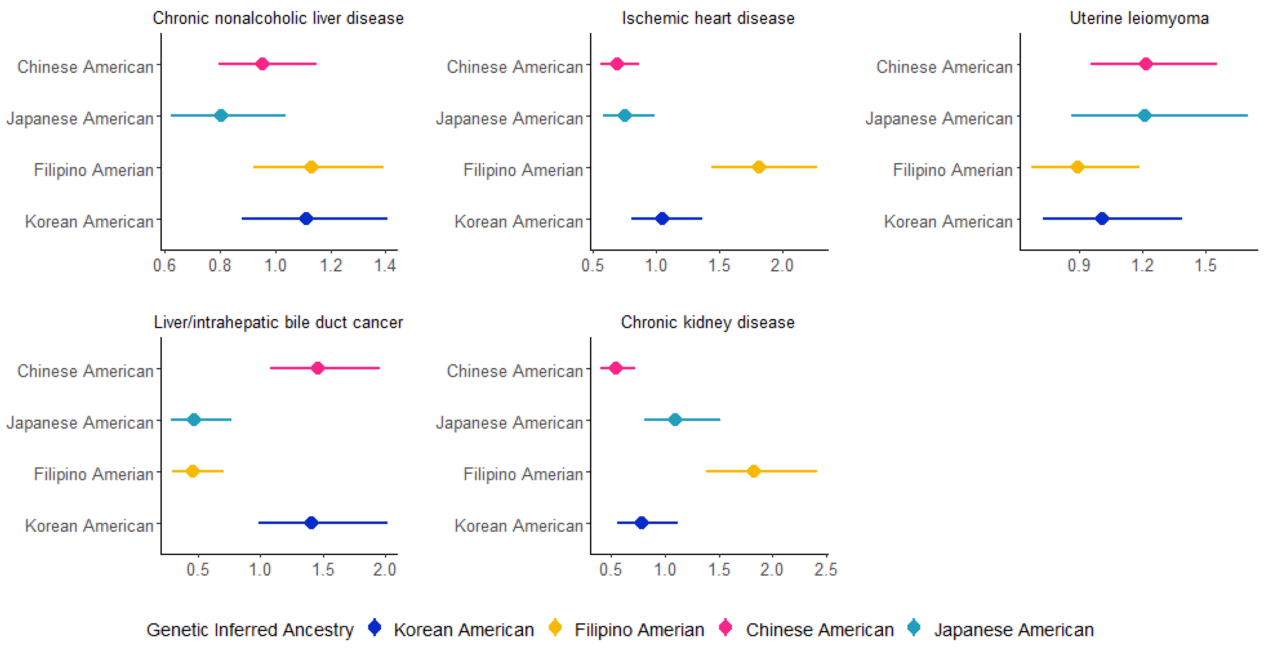

Fig. S10: Disease associations vary across subcontinental groups within the East Asian American GIA group. For individuals in the East Asian American GIA group in ATLAS (N=3,331), we show the odds ratio computed from associating each phenotype with individuals' subcontinental GIA group under a logistic regression model. We limit analyses to phenotypes with N>20 cases; for this reason, the analysis for skin cancer has been omitted. Error bars represent 95% confidence intervals.

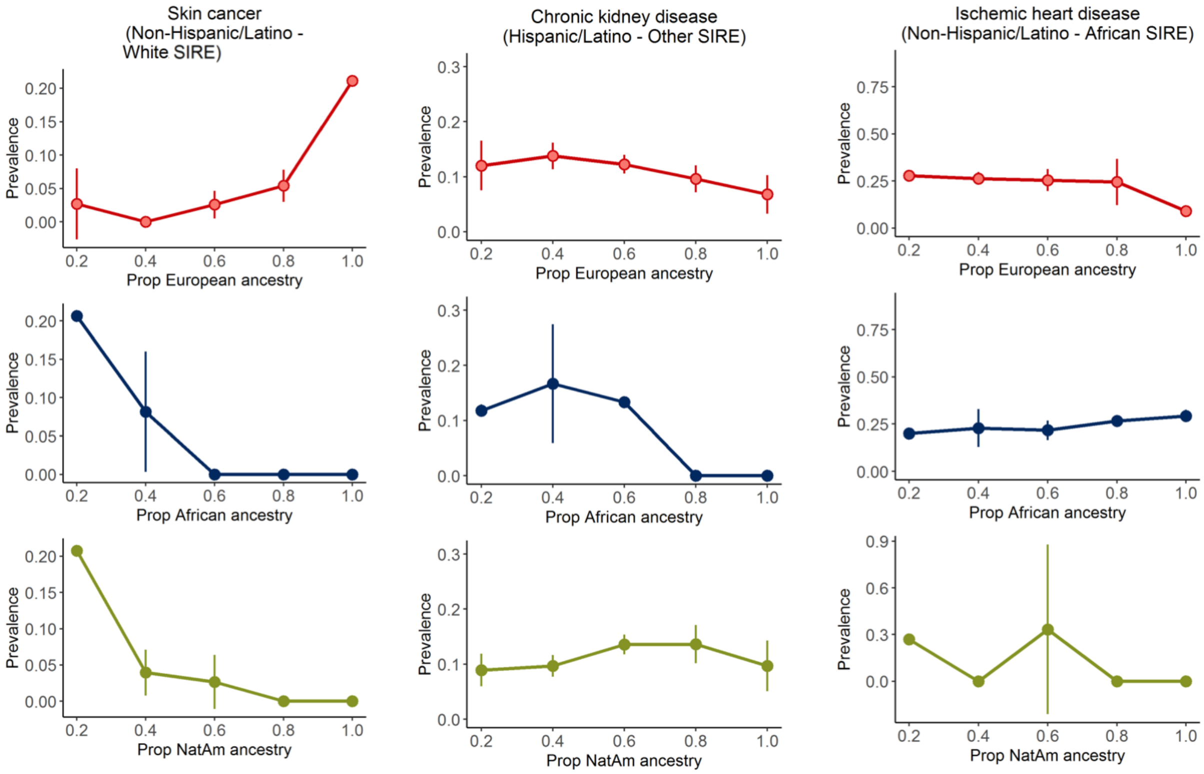

Fig. S11: Global ancestry correlates with disease prevalence in admixed individuals. Individuals by SIRE who have had a diagnosis of (A) skin cancer, (B) chronic kidney disease, or (C) heart disease are binned by their proportions of either European, African, or Native American ancestry estimated using ADMIXTURE. Within each bin, we plot the prevalence of the diagnoses and provide standard errors (+/-1.96 SE) of the computed frequencies.

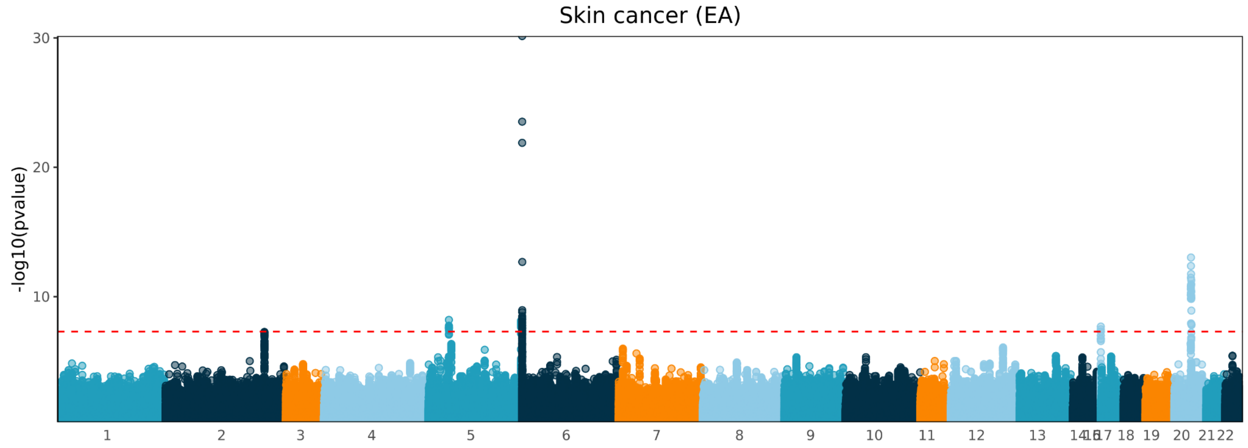

Fig. S12: Manhattan plot for ancestry-specific analysis for skin cancer. GWAS Manhattan plot for skin cancer in the European American GIA group. The red dashed line denotes genome-wide significance (*p*-value<5×10^{-8}).

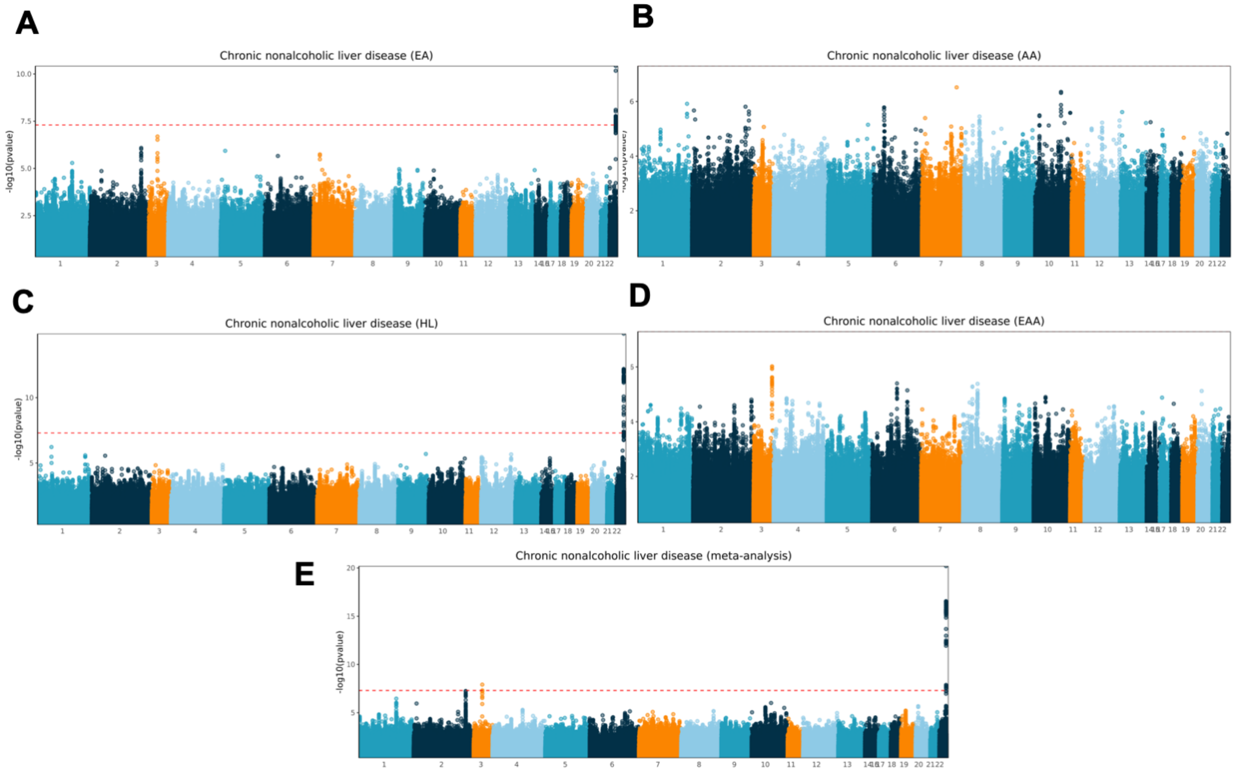

Fig. S13: Manhattan plots for ancestry-specific and multi-ancestry meta-analysis for chronic nonalcoholic liver disease. GWAS Manhattan plots for chronic nonalcoholic liver disease in the (A) European American, (B) African American, (C) Hispanic Latino American, (D) East Asian American GIA groups, and (E) the meta-analysis across all GIA groups. The red dashed line denotes genome-wide significance (p-value$<5\times 10^{-8}$).

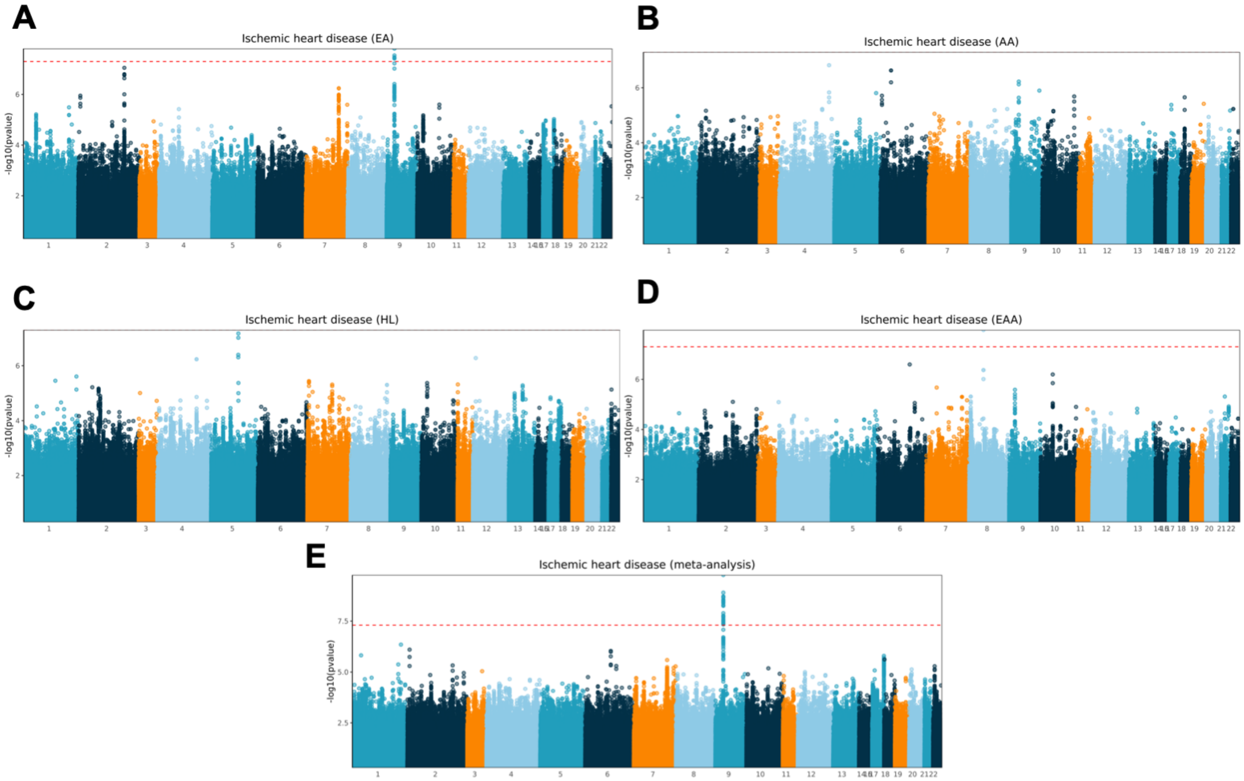

Fig. S14: Manhattan plots for ancestry-specific and multi-ancestry meta-analysis for ischemic heart disease. GWAS Manhattan plots for ischemic heart disease in the (A) European American, (B) African American, (C) Hispanic Latino American, and (D) East Asian American GIA groups, and (E) the meta-analysis across all GIA groups. The red dashed line denotes genome-wide significance (p-value$<5\times 10^{-8}$).

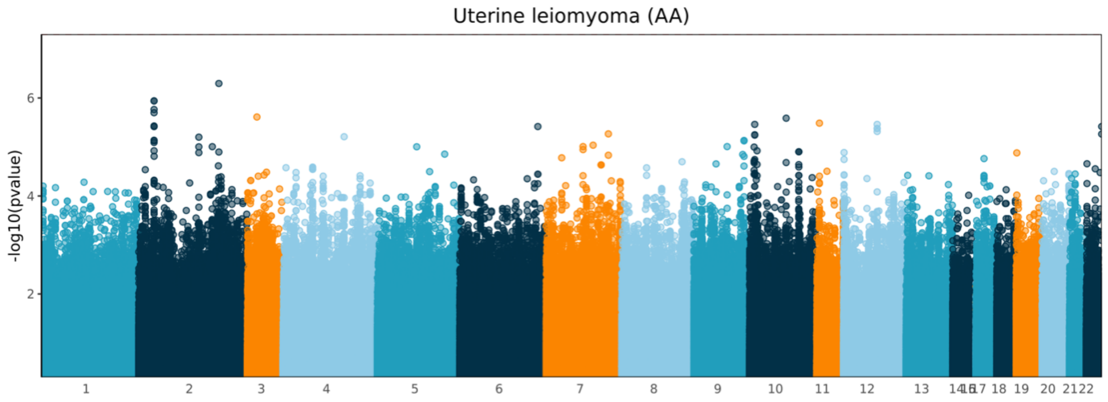

Fig. S15: Manhattan plot for ancestry-specific analysis for uterine leiomyoma. GWAS Manhattan plots for uterine leiomyoma in the African American GIA group. The red dashed line denotes genome-wide significance (p-value<5×10^{-8}).

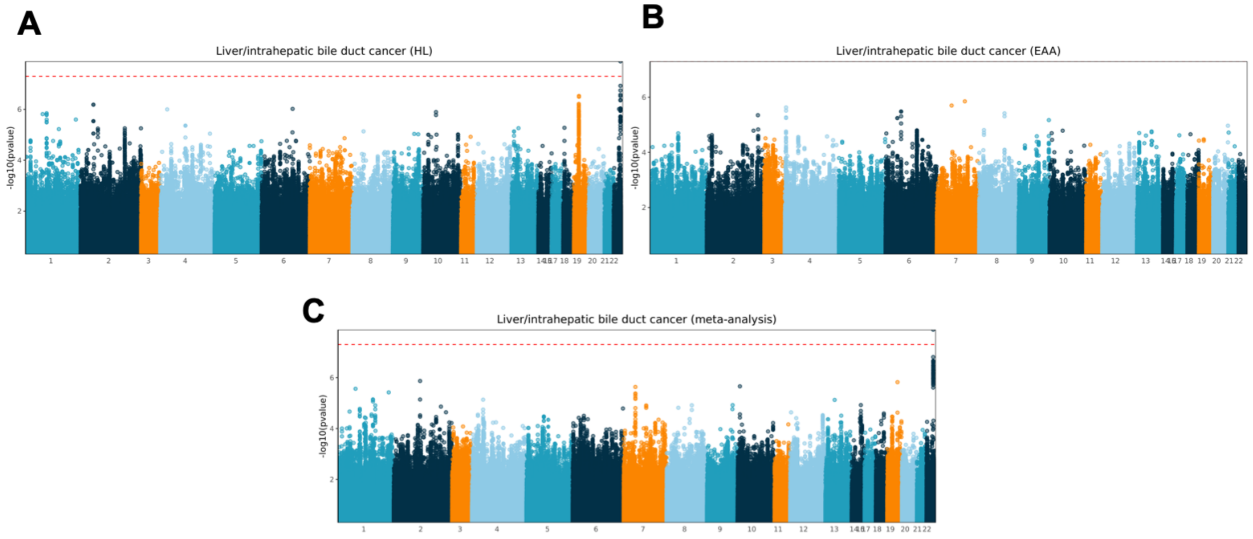

Fig. S16: Manhattan plots for ancestry-specific and multi-ancestry meta-analysis for liver/intrahepatic bile duct cancer. GWAS Manhattan plots for liver/intrahepatic bile duct cancer in the (A) Hispanic Latino American, (B) East Asian American GIA groups, and (C) the meta-analysis across both GIA groups. The red dashed line denotes genome-wide significance (*p*-value<5×10^{-8}).

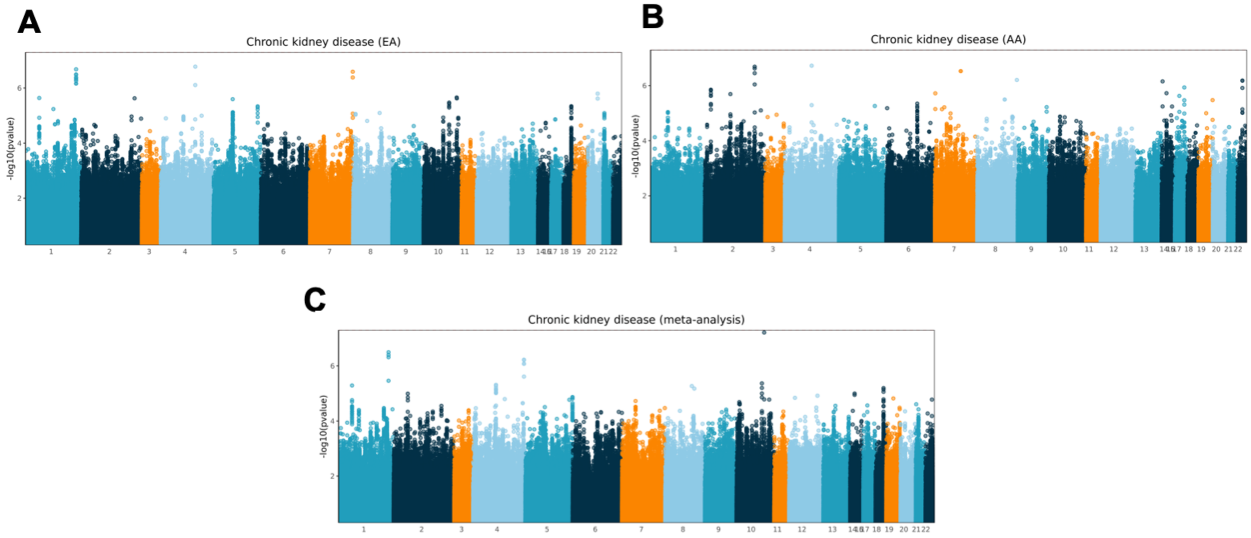

Fig. S17: Manhattan plots for ancestry-specific and multi-ancestry meta-analysis for chronic kidney disease. GWAS Manhattan plots for chronic kidney disease in the (A) European American, (B) African American, and (C) the meta-analysis across GIA groups. The red dashed line denotes genome-wide significance (p-value$<5\times 10^{-8}$).

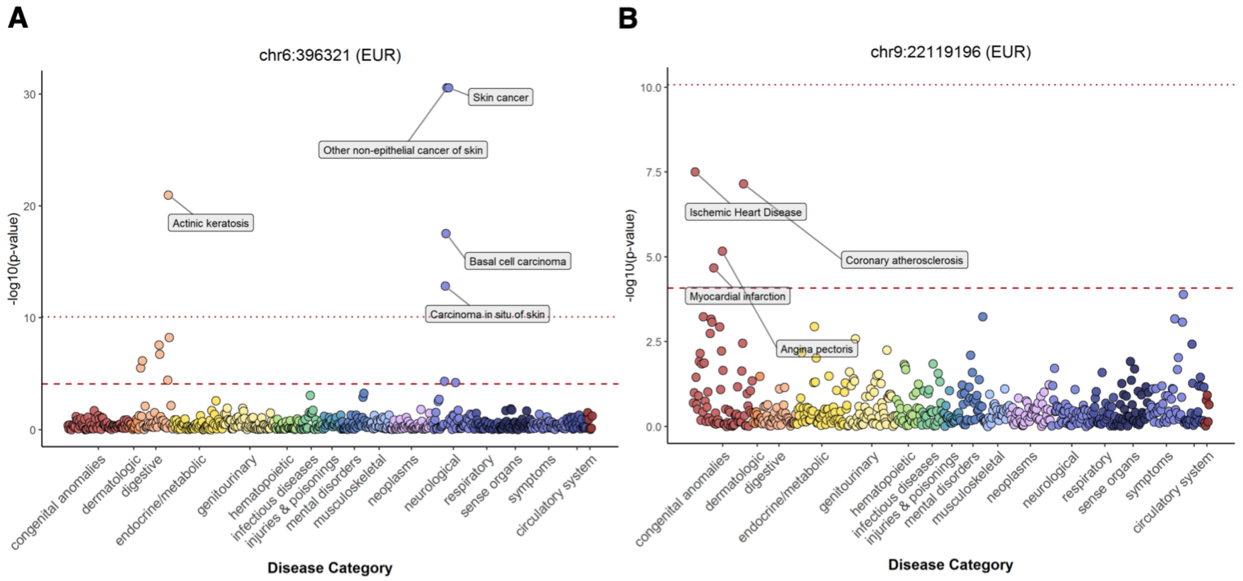

Fig. S18: PheWAS at top GWAS associations. We show a PheWAS plot at rs12203592 (chr6:396321) and rs1333045 (chr9:22119196) computed within the European American GIA group. The red dashed line denotes p-value=4.09× 10^{-5}, the significance threshold after adjusting for the number of tested phenotypes. The red dotted line denotes the significance threshold after correcting for both genome-wide significance and the number of tested phenotypes (p-value=4.09× 10^{-11}).

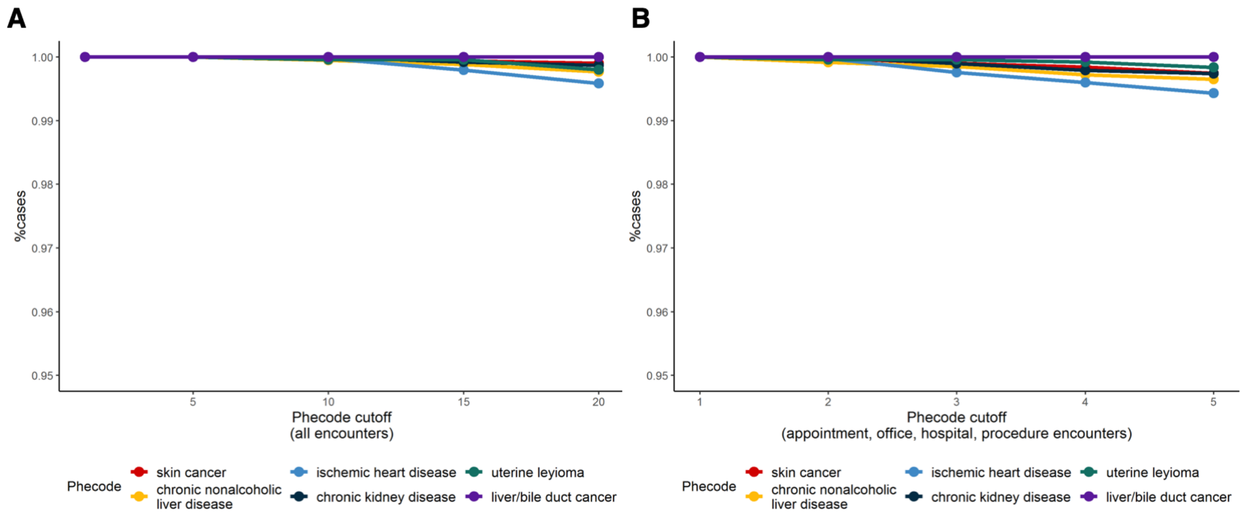

Fig. S19: Role of phecode occurrences for defining cases. We show the percentage of cases retained while varying the number of required phecode occurrences (x-axis) for 6 phenotypes. In A), phecodes are derived from all types of encounters. In B), phecodes are only derived only from appointments and office, hospital, or procedure visits. The y-axis ranges from 0.95 to 1.0.
